# Modeling Influenza Antiviral Strategies: Reducing Burden and Preventing Resistance

**DOI:** 10.1093/infdis/jiaf263

**Published:** 2025-10-17

**Authors:** Remy Pasco, Frederick G Hayden, Lauren Ancel Meyers

**Affiliations:** Integrative Biology, The University of Texas at Austin, Austin, Texas, USA; Department of Medicine, University of Virginia School of Medicine, Charlottesville, Virginia, USA; Integrative Biology, The University of Texas at Austin, Austin, Texas, USA; Santa Fe Institute, Santa Fe, New Mexico, USA

**Keywords:** influenza, antiviral, drug resistance, public health, infectious diseases

## Abstract

Annually, influenza epidemics lead to hundreds of thousands of deaths worldwide many more hospitalizations. The antivirals baloxavir and oseltamivir improve outcomes and limit virus spread, but their widespread use may accelerate the emergence of drug-resistant influenza variants, particularly in young children. Using a data-driven model, we assess various age-stratified antiviral treatment strategies, in terms of reducing both illness and the risk of resistance. In a typical influenza season in the United States, administering baloxavir to 20% of symptomatic individuals >5 years old would be expected to reduce the median disability-adjusted life-years (DALYs) lost by 32.3%, but with a 26.4% risk of resistance transmitting widely. If those patients instead received oseltamivir, DALYs lost would decrease by 19.5%, with only a 5.4% chance of widespread resistance. Strategies such as suspending baloxavir use on detection of resistance or administering combination baloxavir-oseltamivir therapy could further mitigate these risks. For example, treating the same patient population with combination therapy would achieve a 33.5% reduction in DALYs lost, with a 10.2% likelihood of baloxavir resistance emergence and community transmission.

Seasonal influenza remains a major cause of disease and death globally, leading to an estimated 290 000–650 000 deaths each year [1] and causing many more people to be hospitalized. Annual vaccination campaigns help reduce that burden but are impeded by low coverage (<50% of people receive the vaccine in a typical season [2]) and limited vaccine effectiveness due to the rapid evolution of the influenza virus, which makes it challenging to match vaccine strains to circulating strains [3, 4]. Antiviral drugs are used both therapeutically to reduce illness duration and complications and, much less frequently, prophylactically to prevent illness, particularly in high-risk individuals. If taken shortly after the onset of symptoms, antivirals can reduce infectiousness and thereby reduce virus transmission to individuals who come in contact with treated case patients [5, 6]. In addition to mitigating the seasonal burden of influenza, antivirals are stockpiled by the United States and other nations as a first line of defense against novel influenza viruses that spill over from wild or domesticated animals, such as the highly pathogenic avian influenza A(H5N1) observed in humans in the United States in 2024 [7, 8].

Two classes of antivirals are currently recommended for influenza treatment in the United States: the neuraminidase inhibitors, including the most commonly prescribed antiviral oseltamivir which is approved for all ages (brand name Tamiflu), and the endonuclease inhibitor baloxavir marboxil, approved for adults and children aged ≥5 years (brand name Xofluza; referred to as *baloxavir* hereafter) [9]. In Europe, baloxavir is approved for treatment and prevention of influenza starting at 1 year of age [10]. Baloxavir and oseltamivir are effective in reducing the duration of symptoms in patients treated for influenza A virus illness, but clinical trial data indicate that upper respiratory tract infectious viral titers decrease faster in baloxavir-treated than oseltamivir-treated patients [11]. Findings from prior observational studies and a randomized, placebo-controlled trial of oseltamivir treatment indicate that its timely use may reduce virus transmission to household contacts [12, 13]. Observational data indicate that baloxavir treatment also reduces the risk of onward virus transmission to close contacts [6], and a randomized placebo-controlled trial measured this effect [14, 15].

Assuming that the infectiousness of baloxavir-treated patients declines in proportion to their upper respiratory tract viral titers, a modeling study estimated that increasing the use of baloxavir for treating infected adults within 48 hours of symptom onset could substantially reduce the overall burden and economic costs of seasonal influenza epidemics [5]. An individual's infectiousness refers to their ability to transmit influenza to susceptible people they encounter; it can vary during the course of infection, depending on viral load and the duration of illness, and the individual’s total infectiousness is their overall ability to infect others throughout the infectious period.

However, broad use of antivirals might promote the emergence of antiviral-resistant variants [16]. The impact of such variants on individuals and populations depends on their replicative fitness, virulence, and transmissibility relative to drug-susceptible, wild-type strains. Adamantane-resistant influenza A viruses have been observed to emerge both spontaneously and during antiviral treatment [17]. Transmission of these viruses was first documented in household settings [18] and subsequently detected globally, prompting the suspension of adamantane-class antivirals for seasonal influenza A [19]. Resistant variants may also emerge in the apparent absence of selective drug pressure. For example, a new strain of seasonal influenza A(H1N1) with highly reduced susceptibility to oseltamivir emerged during the 2007–2008 influenza season and then spread globally [20, 21], until it was replaced by the oseltamivir-susceptible pandemic 2009 A(H1N1) virus.

For baloxavir, the emergence of variants with reduced susceptibility has been observed in clinical trials of both immunocompetent adults and children [11, 22], especially those being treated for influenza A(H3N2) virus illness [11]. Although human transmission of influenza A(H3N2) viruses with reduced susceptibility to baloxavir have been observed [23, 24], preclinical trial findings suggest that variants of both A(H3N2) and A(H1N1) carrying the polymerase acidic I38T substitution (which confers lower susceptibility) have diminished within-host and between-host fitness relative to the wild type [25, 26].

As part of its national influenza surveillance programs, the US Centers for Disease Control and Prevention (CDC) monitors genetic changes associated with resistance to antivirals [27] and guides clinicians accordingly regarding antiviral use [9]. When baloxavir was approved for use in the United States, the CDC validated and implemented new assays for molecular markers of baloxavir resistance in laboratories across the country [28, 29].

In the current study, we used a mathematical model that links within-host viral replication dynamics to between-host transmission to estimate the impact of expanding antiviral treatment with baloxavir, oseltamivir, or their combination (henceforth, *combination therapy*) on both the overall burden of disease and the risks of antiviral resistance transmission at the population level during a typical influenza season in the United States.

## METHODS

We first estimate the infectiousness profile of individuals infected with influenza by fitting a previously published within-host model to clinical trial data from recent years [5, 11, 22, 30]. We then use a transmission model to simulate influenza epidemics and assess the impact of various antiviral treatment strategies on both disease burden and the spread of antiviral resistance.

### Within-Host Influenza Viral Dynamic Model

Following the methods of reference [5], we model the within-host replication dynamics of antiviral-sensitive influenza viruses using stochastic compartmental models, one for adults and one for children [31]. The models are initially described by a set of deterministic ordinary differential equations (Supplementary Appendix, Section 1.1), which are fit to viral titer data from 1014 adult and 190 children patients with influenza who received either baloxavir, oseltamivir, or a placebo [11, 22, 32] (Supplementary Table 1.4). We assume that combination baloxavir-oseltamivir therapy has the same efficacy as baloxavir-alone therapy for patients infected with baloxavir-susceptible virus and the same efficacy as oseltamivir-alone therapy for patients infected with baloxavir-resistant virus, based on clinical trial results [30, 33].

For antiviral-resistant influenza viruses, we make the more rudimentary assumption that viral load increases linearly to a peak that occurs a few days after the virus becomes detectable, and then decreases linearly to zero (Supplementary Appendix, Section 1.2), matching the dynamics observed in previous clinical trials [34–38]. The model uses published estimates for the probability of resistance emergence following treatment, depending on the infecting subtype, influenza A(H1N1) or influenza A(H3N2) [11, 22, 38] (Table 1).

**Table 1.** Probability of Antiviral-Resistant Variant Emergence Following Treatment With Baloxavir, Oseltamivir, or the Combination of Both

Patient Age Group	Probability of Drug-Resistant Variant Emergence During Treatment^a^					
	Oseltamivir^b^		Baloxavir^b^		Combination Therapy^c^ (Baloxavir Resistance)	
	A(H1N1)	A(H3N2)	A(H1N1)	A(H3N2)	A(H1N1)	A(H3N2)
0–4 y	16.1	7.7	14.1	23.8	6.0	10.1
5–17 y	2.8	2.1	6.6	11.1	2.8	4.7
≥18 y	1.7	0.8	2.2	9.7	0.9	4.1

^a^Probabilities depend on the treated individual's age group and the circulating influenza subtype and are expressed as percentages. Treatment with combination therapy is assumed to potentially produce baloxavir resistance but not oseltamivir resistance

^b^Based on published estimates for oseltamivir [38] and baloxavir [11] resistance risks in adults and baloxavir resistance risks in children [22].

^c^Derived by scaling published estimates for baloxavir resistance risks by the risk reduction measured in a clinical trial of combination therapy in adults [30].

We assume that infectiousness is logarithmically proportional to upper respiratory tract viral titer, based on prior analysis linking viral load to secondary infection risk [39]. For antiviral-resistant viruses, we assume that viral titer dynamics within a host are the same as for sensitive viruses but that the corresponding infectivity may be reduced, depending on the assumed fitness cost of resistance.

### Between-Host Influenza Transmission Model

We use a stochastic age- and risk-stratified individual-based model of influenza transmission to simulate the simultaneous transmission of wild-type, baloxavir-resistant, and oseltamivir-resistant influenza viruses and age-specific antiviral treatment strategies throughout the course of a single influenza season. We use the fitted within-host model to parametrize the daily infectiousness of infected children and adults, depending on the infecting virus and the type and timing of antiviral treatment (if received). Antivirals are administered to a fraction of symptomatic patients. We assume that the time between symptom onset and treatment is ≤48 hours and is distributed according to estimates from clinical trial data [11, 36] (Supplementary Table 2.5). Antivirals are assumed to lower the risks of hospitalization and death, at age- and risk group–specific efficacies [40], and to reduce daily infectiousness according to the within-host model (Supplementary Table 2.5).

We calibrate our base scenario so that the wild-type seasonal virus has a basic reproductive number (*R*_0_) of 1.28 [41]. For antiviral-resistant variants, *R*_0_ is reduced by 0%–15%, depending on the assumed fitness cost. As a sensitivity analysis, we analyzed pandemic scenarios with an *R*_0_ of 2.5 and milder seasonal influenza scenarios with an *R*_0_ of 1.12 (Supplementary Appendix, Sections 4.8 and 4.9). Antiviral-resistant viruses can only arise following treatment, with probabilities that depend on the age of the patient and the virus subtype.

### Antiviral Treatment Strategies

We analyze 29 treatment strategies during typical A(H1N1)-dominant and A(H3N2)-dominant influenza seasons in the United States, separately. First, we compare the impacts of oseltamivir-only, baloxavir-only and combination therapy across symptomatic treatment rates ranging from 0% to 40% in 10% point increments. Then we fix the treatment rate to 20% and evaluate a range of age group–specific and risk group–specific treatment policies. Finally, we consider surveillance-based triggers for suspending baloxavir and combination therapy when baloxavir-resistant infections exceed 5% of the weekly incident infections. Appendix Section 4.5 provides results for analogous oseltamivir triggers and explores alternative thresholds.

### Outcome Measures

We compare antiviral treatment strategies for both public health benefits and risk of promoting the emergence and spread of antiviral-resistant viruses. To compare benefits, we estimate the disability-adjusted life-years (DALYs) lost over a typical influenza season according to methods published in [42] (Appendix Section 3). The DALY metric estimates the burden of disease in terms of years of life affected by disability due to infection or hospitalization and the years of life lost due to premature death from infection [43], with oseltamivir and baloxavir both improving all metric components. DALYs are widely used in public health because they combine different health outcomes into a single metric, allowing comparisons of the total burden of disease across various interventions and age groups. To compare risks, we estimate the probability that baloxavir-resistant viruses will reach a prevalence of ≥20% of all infections at some point during the influenza season.

## RESULTS

**Figure 1.**
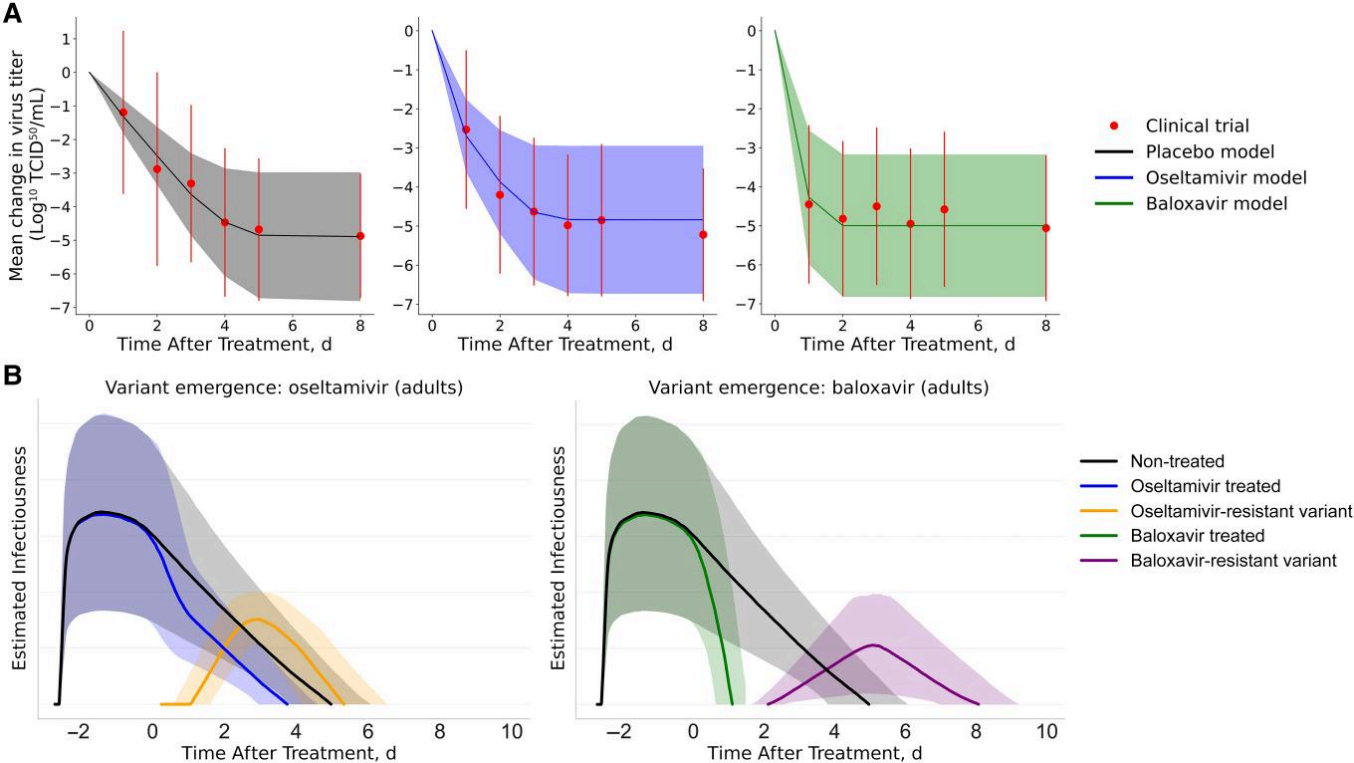
Estimated influenza viral titers and infectiousness trajectories, for treated and untreated influenza infections in adults. Corresponding figures for children are provided in the Supplementary Appendix, Section 1.1. *A*, Means and standard deviations of wild-type influenza titers across simulated patients (*lines and shading*) and clinical trial participants (*dots and error bars*), for untreated (*left*), oseltamivir-treated (*middle*) and baloxavir-treated (*right*) patients. Abbreviation: TCID_50_, median tissue culture infective dose. *B*, Model-estimated infectiousness of untreated cases (*black lines and shading*), oseltamivir-treated cases (*left graph*; *blue line and shading*), and baloxavir-treated cases (*right graph*; *green line and shading*), as a function of days since treatment (day 0). The y-axes represent relative infectiousness, scaled to achieve the specified population-level *R*_0_ for each scenario analyzed. For the small fraction of patients who develop antiviral resistance (Table 1), the orange and purple curves indicate the estimated infectiousness of the resistant virus. Lines represent medians; shading, 95% confidence intervals estimated from 2000 stochastic simulations. Estimates assume that treatment is initiated 12–24 hours after symptom onset and that infectiousness is logarithmically related to viral load.

Baloxavir inhibited viral replication similarly in adults and children and significantly faster than oseltamivir (Figure 1, Supplementary Appendix, Sections 1.1 and 1.3, and Supplementary Table 1.4). During the first 24 hours following the initiation of baloxavir treatment, our model projects an average decrease in upper respiratory tract viral titer of 95% (95% confidence interval [CI], 44%–100%) in adults and 88% (83%–100%) in children; for oseltamivir and placebo, the corresponding decreases are 31% (24%–35%) and 10% (5%–18%) in children and 44% (37%–47%) and 25% (19%–34%) in adults, respectively. For both age groups, we estimate that baloxavir treatment accelerates the nearly complete elimination of viral load by roughly 2 days relative to oseltamivir treatment and ≥3 days relative to no treatment.

Clinical data suggest that antiviral-resistant variants are generally more likely to arise during baloxavir than during oseltamivir treatment and that the risks are higher for young children than for adolescents and adults (Table 1). When this occurs during oseltamivir treatment, the resistant viruses are first detected an average of 2.3 days after symptom onset for adults and 2.7 days after onset for children, typically while wild-type virus remains at detectable levels [38]. For baloxavir-resistant variants, antiviral-resistant variants tend to fully replace the wild-type virus so that the latter is no longer detectable, an average of 2.9 and 3.5 days after symptom onset for adults and children, respectively [34–37].

**Figure 2.**
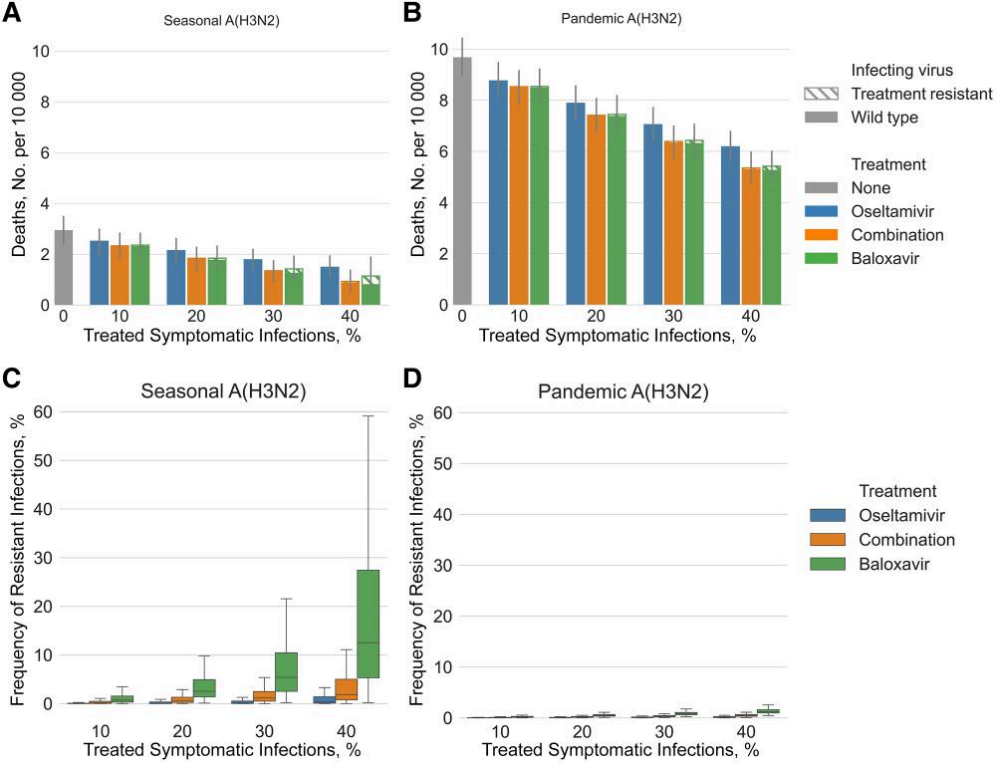
Projected impact of expanding treatment with oseltamivir, baloxavir, or combination therapy for seasonal and pandemic influenza A(H3N2) virus. *A, B,* For a seasonal virus (*A*) with wild-type reproduction number (*R*_0_) of 1.28 and antiviral-resistant *R*_0_ of 1.15, and a pandemic virus (*B*) with wild-type *R*_0_ of 2.5 and antiviral-resistant *R*_0_ of 2.25, bar heights indicate median deaths per 10 000 resulting from infection with a wild-type (*solid bars*) or antiviral-resistant (*hatched bars*) virus, based on 500 stochastic simulations. Gray lines indicate 95% confidence intervals and x-axes, the proportion of symptomatic patients who receive treatment; colors correspond to the type of treatment administered. *C, D,* Estimated proportions of infections caused by the transmission of antiviral-resistant variants in the seasonal (*C*) and pandemic (*D*) scenarios. Analogous results for the influenza A(H1N1) subtype and for antiviral-resistant variants with higher or lower fitness are provided in the Supplementary Appendix, Section 4.4.

We first estimate the impact of expanding baloxavir, oseltamivir, or combination therapy treatment for all age groups in the United States during an influenza A(H3N2) season with a basic reproduction number of 1.28 and during an influenza A(H3N2) pandemic with a basic reproduction number of 2.5 (Figure 2), assuming that antiviral-resistant viruses have 10% lower transmission rates than their corresponding wild-type strain. This 10% reduction is informed by several in vivo ferret studies evaluating the fitness of baloxavir- and oseltamivir-resistant viruses [44, 45]. Those studies consistently found small but unquantified reductions in competitive fitness. In addition, permissive mutations enabling antiviral resistance without significant fitness loss have been documented, as with the seasonal oseltamivir-resistant A(H1N1) viruses that started circulating in 2007 until being replaced by pH1N1 in 2009 [46]. Additional sensitivity analyses exploring alternative reductions are provided in the Appendix.

We provide results for similar influenza A(H1N1) scenarios in the Appendix, Section 4.2. Treating 20% of symptomatic individuals reduces the expected proportion of the population infected by 11.9% (95% CI, .6%–22.6%) with oseltamivir, 20.1% (8.9%–32.6%) with baloxavir, and 20.6% (9.6%–31.9%) with combination therapy. The overall mortality rate is expected to decline by 26.2% (95% CI, 7.5%–43.2%), 36.7% (18.7%–52.0%), and 37.5% (19.0%–54.0%) for oseltamivir, baloxavir, and combination therapy, respectively (Figure 2*A*). The reductions in mortality rate are much greater than the reductions in infections, as antivirals reduce both total infections and the chance of death given infection. When 20% of symptomatic people are treated with baloxavir, 54.8% of the reduction in mortality rate is achieved by averting infections in the first place, and another 45.2% corresponds to deaths averted by treatment of infected individuals. As we increase the treatment rate, we project significantly lower expected burden of disease yet higher prevalence of antiviral-resistant viruses.

Mass antiviral treatment is expected to reduce a greater number but a smaller proportion of influenza-related deaths in the pandemic scenario than in the seasonal epidemic scenario (Figure 2*A* and 2*B*). Treatment of 20% of symptomatic cases with baloxavir is expected to reduce pandemic deaths by 23.0% (95% CI, 14.1%–30.9%), and using oseltamivir or combination therapy instead is expected to reduce influenza deaths by about 18.3% (9.7%–26.6%) or 23.2% (15.2%–30.8%), respectively. However, the risk that antiviral-resistant viruses will emerge and spread widely is significantly lower in the pandemic than in the seasonal influenza scenario (Figure 2*C* and 2*D*), even for a resistant variant with fitness equal to the wild type (Supplementary Figure 4.9). The higher pandemic transmission rate of wild-type virus causes a more rapid depletion of susceptible hosts, which impedes the emergence of resistant variants. Similar results for treatment of specific age and risk groups under a variety of influenza A(H3N2) and A(H1N1) scenarios are provided in Supplementary Figure 4.3.

**Figure 3.**
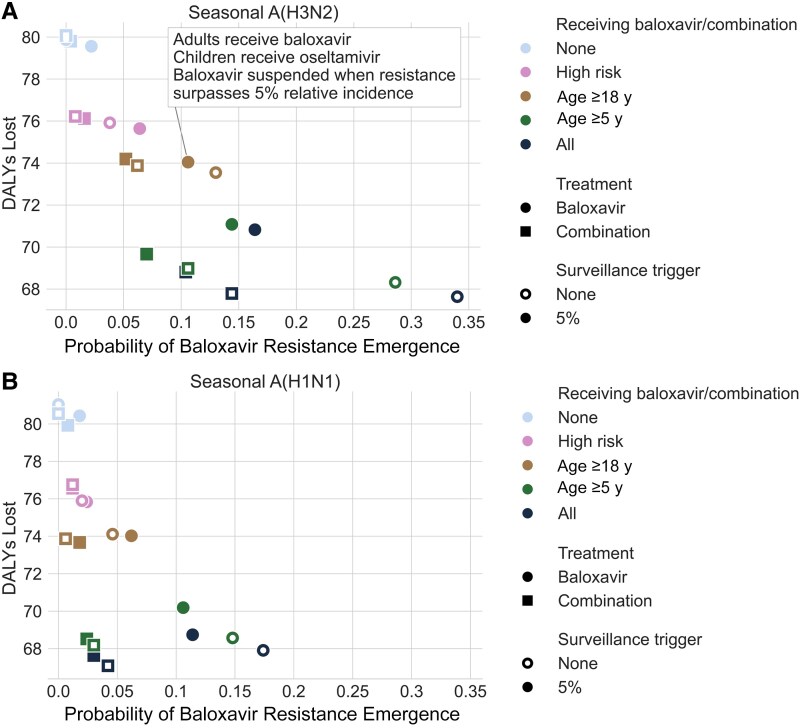
Comparative evaluation of oseltamivir, baloxavir, and combination antiviral treatment strategies for seasonal influenza epidemics, shown as the trade-off between public health benefits of antiviral treatment (disability-adjusted life-years [DALYs] lost per 10 000 individuals) and the risk that antiviral-resistant viruses will emerge and spread (probability of baloxavir-resistant infections exceeding 20% of incident infections anytime during the season), for influenza A(H3N2)–dominant (*A*) and influenza A(H1N1)–dominant (*B*) seasons. Each of the 20 antiviral treatment strategies assumes that 20% of symptomatic patients receive treatment and specifies the following treated subpopulations to receive either baloxavir (*circles*) or combination therapy (*squares*) rather than oseltamivir: none (*light blue*), high-risk individuals only (*pink*), adults aged >18 years (*brown*), children and adults aged >5 years (*green*), or all patients (*black*). Each strategy also either does (*filled symbols*) or does not (*open symbols*) include a surveillance-based trigger for suspending baloxavir and combination therapy when baloxavir-resistant viruses surpass 5% relative incidence and for suspending oseltamivir when oseltamivir-resistant viruses surpass 5% relative incidence. The estimates are medians across 500 stochastic simulations.

We compare several age-specific treatment strategies in terms of both burden averted and baloxavir resistance risks, assuming that 20% of symptomatic patients receive treatment (Figure 3). In a baseline scenario of oseltamivir-only treatment for all age groups, we would expect that 441 per 10 000 people in the United States would receive treatment, resulting in 22.6 (95% CI, 18.9–26.8) or 22.8 (19.3–26.7) influenza-related hospitalizations, respectively, per 10 000 people over a typical influenza A(H3N2)–dominant or influenza A(H1N1)–dominant influenza season. These correspond, respectively, to 79.6 (95% CI, 68.0–93.2) or 80 (69.2–92.4) DALYs per 10 000 lost due to seasonal influenza. If all treated patients receive baloxavir rather than oseltamivir, then we would expect the DALYs lost per 10 000 to decrease by 14.4% to 68.1 (95% CI, 54.7–81) for A(H3N2) or by 14.9% to 68.1 (54.6–79.0) for A(H1N1), while the risk of baloxavir-resistant viruses reaching a relative frequency of 20% would rise from 0% to 35.4% or 16.6%, respectively (Figure 3*A* and 3*B*, *black open circles*). Replacing baloxavir monotherapy with combination therapy would reduce the emergence risks to 14.4% for A(H3N2) and 4.2% for A(H1N1) (Figure 3*A* and 3*B*, *black open squares*).

Age-restrictive treatment strategies can balance the costs and benefits of treatment. Treating adults (aged >18 years) with baloxavir and children with oseltamivir would be expected to result in 73.5 (95% CI, 62.8–86.0) or 74.1 (60.9–84.9) DALYs lost per 10 000 in A(H3N2)-dominant or A(H1N1)-dominant seasons, respectively, while reducing the probability of baloxavir-resistance emergence to 13.0% and 4.6%, respectively (Figure 3*A* and 3*B*, *brown open circles*). For an A(H3N2) season in which baloxavir resistance does arise, we estimate a 7.5% chance that it will surge before the seasonal peak (Supplementary Figure 4.7). Incorporating a fail-safe trigger, which suspends the use of an antiviral once resistant viruses exceed 5% relative incidence, further reduces the estimated probability of emergence to 10.6% for an A(H3N2) season without significant increasing the estimated DALYs lost (Figure 3*A*, *brown filled circles*). Administering combination therapy rather than baloxavir alone is expected to significantly reduce emergence risks without increasing hospitalizations, across all strategies considered (Figure 3*A* and 3*B*, *squares*).

## DISCUSSION

Influenza antivirals, when administered to symptomatic patients shortly after symptom onset, can shorten the duration of illness, reduce complications, and lower the risks of transmission. Consequently, expanding the use of timely antiviral treatment is anticipated to lessen the overall societal burden of influenza and ease the strain on healthcare systems during severe epidemics [5, 47, 48]. However, the widespread use of antivirals can promote the emergence and spread of antiviral-resistant variants, which could compromise the effectiveness of these drugs. Clinical studies show that baloxavir reduces viral shedding more rapidly and complications to greater extent than oseltamivir [49] yet may carry a higher risk of antiviral resistance, especially for H3N2 viruses [11].

Despite this concern, oseltamivir-resistant variants have not spread widely since 2008 [21], and baloxavir-resistant variants have not yet emerged on a large scale [50]. A clinical trial evaluating baloxavir postexposure prophylaxis in households, where both influenza A(H3N2) and A(H1N1) subtypes circulated, reported that none of the 152 baloxavir-treated index patients transmitted a baloxavir-resistant variant to their household contacts [51], and no transmission of baloxavir-resistant variants from treated index patients to household contacts was detected in the recently completed CENTERSTONE trial [14, 15]. Limited transmission of baloxavir-resistant influenza virus has been detected in Japan, where only a single community cluster among adults has been reported [23, 24, 52]. During the first season of baloxavir use in Japan (2018–2019), up to 5.3 million of roughly 12.0 million total infected individuals received baloxavir. The Japanese national influenza surveillance system reported that 8.0% of assayed influenza A(H3N2) viruses and 2.3% of assayed influenza A(H1N1) viruses that season were resistant to baloxavir [53]. In all subsequent seasons through 2023–2024, the proportion of both A(H3N2) and A(H1N1) viruses resistant to baloxavir has remained below 2.0% in Japan, and the proportion of antiviral-resistant viruses detected in the United States has also been well below 1.0% in the past few seasons [29], which is consistent with our model projections and suggests that these variants may have lower transmission fitness relative to the wild type.

We have identified 2 antiviral strategies that can reduce burden while mitigating resistance risks: limiting baloxavir to adults only or using it in a combined regimen with oseltamivir. In a typical A(H3N2)-dominant season, we estimate that treating 20% of all children and adults with baloxavir would be expected to reduce influenza-associated deaths by 37.9% (for oseltamivir, the corresponding estimated reduction is 27.6%) but would run a 34.0% risk of fueling widespread baloxavir resistance. Restricting baloxavir to adults while using oseltamivir for children slightly lowers the benefits (33.0% reduction in mortality rate) while dramatically reducing the risk of resistance emergence (13.0%).

Administering combination therapy instead of baloxavir monotherapy in adults provides the same expected reduction in mortality rate while further lowering the resistance risks (6.2%). For resistance variants with higher fitness, both the risks of emergence following baloxavir monotherapy and the risk-reduction benefits of combination therapy would be higher (Supplementary Appendix, Section 4.10). While costlier than monotherapy, combination therapy might allow dose sparing, potentially reducing oseltamivir adverse effects and offsetting some of the additional costs [54]. However, the viability, safety, and effectiveness of this dose-sparing strategy have not yet been evaluated in clinical trials. Importantly, the expected benefits and risks of any antiviral strategy depend not only on the age-specific treatment rates and protocols, but also on the dominant influenza subtype, its transmission rate, and the relative fitness of any emerging resistant variants, which can vary across seasons [55], and regions [56]. Broadening access to rapid influenza diagnostic tests that distinguish between influenza A subtypes [57], may allow clinicians to tailor antiviral use more effectively.

Under a pandemic scenario, we find that mass antiviral campaigns may only modestly reduce transmission yet significantly decrease the mortality rate, while posing a relatively low risk of treatment resistance. Although not analyzed here, antivirals could also be administered prophylactically to further limit transmission and lower the overall incidence and mortality rate. Key challenges to such strategies include maintaining sufficient stockpiles and ensuring rapid, equitable, and effective distribution. Given that antivirals may be the only effective medical countermeasure prior to vaccine availability during an emerging influenza pandemic, the potential benefits may far outweigh the stockpiling costs.

Our results underscore the need for effective virologic surveillance. Early detection of antiviral-resistant variants is critical to curbing widescale transmission but may require assaying thousands of influenza positive specimens each week, depending on the desired speed, precision, geographic scale of detection [58], and sequencing technique [29, 59, 60]. The fitness of antiviral-resistant variants may depend on the influenza A subtype and specific drug-related substitution involved. For oseltamivir, resistance is more commonly reported in A(H1N1) than in A(H3N2) viruses, with higher replicative capacity observed in vitro and in vivo in A(H1N1) viruses [61]. For baloxavir, the reverse appears true [26]. Although experimental studies suggest that baloxavir-resistant variants may be only slightly less fit than the wild type [26, 44], there have been few case reports of human-to-human transmission [23, 24, 62, 63]. More extensive surveillance, potentially including wastewater sampling [64], can provide vital situational awareness as baloxavir usage expands.

Delays in viral clearance due to treatment-emergent resistant variants are not necessarily correlated with prolonged illness in outpatients but may be associated with poor outcomes in hospitalized adults [11, 30, 38]. Our study did not model this, but combining antivirals with nonpharmaceutical interventions, such as social distancing and face masking, could further reduce the transmission of emerging variants. The effectiveness of such strategies is evident from the reduced seasonal influenza circulation during the early phase of the severe acute respiratory syndrome coronavirus 2 pandemic [65]. However, these strategies have not typically been used to control seasonal influenza in the Western hemisphere, and their usage has been declining [66].

The global spread of oseltamivir-resistant seasonal A(H1N1) virus beginning in 2007 [20], the persistence of adamantane resistance in seasonal influenza A viruses since 2005 [61], our inability to predict dominant influenza strains [67], and the high prevalence of baloxavir resistance in clinical trial patients underscore the pressing need to address the issue of influenza antiviral resistance. Data-driven models can help develop public health strategies that optimize the benefits of novel antivirals in terms of saving lives and reducing costs while also minimizing the risk of resistance, especially during severe influenza outbreaks. However, further surveillance and clinical data are needed to validate and refine such models.

Our model does have limitations. It assumes that infectiousness is logarithmically proportional to viral levels in the upper respiratory tract and does not consider reductions in infectivity due to nonpharmaceutical interventions (eg, quarantine or masking) or illness-related changes in contact patterns and behavior. The risks of baloxavir resistance following combination therapy remain highly uncertain. Dual resistance emergence, observed in certain immunocompromised individuals, may be associated with reduced viral fitness [68]. We do not explicitly model vaccine status, although the assumed reproduction numbers reflect typical levels of vaccine- and infection-acquired immunity during A(H1N1)- and A(H3N2)-dominant seasons. Our simulations assume a single strain of influenza is circulating in a given season. The underlying factors driving variant emergence are not fully understood, but preexisting immunity is thought to play a role [69]. The severity of infections by resistant viruses relative to wild-type infections is largely unknown; here we assume identical case hospitalization and fatality rates.

In conclusion, baloxavir treatment shows considerable promise in reducing influenza burden, but its broad use carries the risk of fueling the spread of resistance. Using a transmission dynamic model that integrates clinical data on antiviral efficacy and resistance emergence, our study compares the cost and benefits of various age-restricted strategies for expanding baloxavir and oseltamivir treatment during severe outbreaks. The findings can inform the design of effective seasonal influenza and pandemic mitigation strategies, the refinement of national molecular surveillance programs, and the stockpiling of antivirals for public health emergencies.

## Supplementary Material

jiaf263_Supplementary_Data

## Supplementary Data

Supplementary materials are available at *The Journal of Infectious Diseases* online (http://jid.oxfordjournals.org/). Supplementary materials consist of data provided by the author that are published to benefit the reader. The posted materials are not copyedited. The contents of all supplementary data are the sole responsibility of the authors. Questions or messages regarding errors should be addressed to the author.

## Notes

***Acknowledgments***. We thank Robert Krug, PhD for his contributions to this manuscript.

***Author contributions***. All authors contributed to the study conception and design. R. P. implemented the model. R. P. and L. A. M. analyzed the data and wrote the first draft. All authors reviewed and contributed to the final draft of the manuscript. L. A. M. supervised the project.

***Financial support.*** This work was supported by the Centers for Disease Control and Prevention Foundation (grant U01IP001136 to R. P. and L. A. M.) and the National Institutes of Health (grant R01 AI151176 to R .P. and L. A. M.).

***Supplement sponsorship***. This article appears as part of the supplement “Advances in Influenza Therapeutics,” sponsored by Flu Lab, World Health Organization, the International Society for Respiratory Viruses, F. Hoffmann-La Roche AG, Shionogi & Co., Ltd., Cidara Therapeutics, Inc., Eradivir Inc, Leyden Laboratories, Gilead Sciences, and the International Federation of Pharmaceutical Manufacturers & Associations.
