# Supplementary material for: Modeling Influenza Antiviral Strategies: Reducing Burden and Preventing Resistance
Source: J Infect Dis. 2025 Oct 17;232(Suppl 3):S299–308. doi: 10.1093/infdis/jiaf263 (PMC12530949; doi:10.1093/infdis/jiaf263)
Supplement: jiaf263_Supplementary_Data [file jiaf263_supplementary_data.pdf]

Supplementary Information for: Modeling influenza antiviral strategies: reducing burden and preventing resistance

Authors: Remy Pasco[a], Frederick G. Hayden[b], Lauren Ancel Meyers[a,c]

[a] Integrative Biology, The University of Texas at Austin, Austin, TX;
[b] Department of Medicine, University of Virginia School of Medicine, Charlottesville, VA;
[c] Santa Fe Institute, Santa Fe, NM

1. Within-host models of viral replication dynamics

We construct separate models to simulate the changing viral titer within an individual following infection with an initial virus and the changing viral titer of a treatment-resistant variant once it arises. Our models assume that influenza A(H1N1) and A(H3N2) infections produce the same viral titer dynamics in infected individuals, but that the two subtypes have different probabilities of evolving treatment resistance (Table 1). We consider only influenza A infections, since the risks of both baloxavir-resistance and oseltamivir-resistance are significantly lower for influenza B infections [1,2].

1.1 Initial infection model

We model the viral kinetics of an individual infected with an influenza A virus following the approach described in [3], with the addition of a latent state *L* after initial infection of cells (as assumed in [4]). The deterministic within-host model is given by the following set of ordinary differential equations:

$$\frac{dU}{dt} = -bUV$$

$$\frac{dL}{dt} = bUV - \gamma L$$

$$\frac{dF}{dt} = \gamma L - \delta F$$

$$\frac{dZ}{dt} = rZ$$

$$\frac{dV}{dt} = (1-\epsilon)pF - cV - kZV$$

where the dynamic variables are:

- U: Susceptible target cells
- L: Cells in a latent state
- F: Infected cells
- Z: Immune response
- V: Free virus

and the parameters are:

- b: Cell infection rate in ml/$TCID_{50}$ per day
- γ: Latent phase rate in $days^{-1}$
- δ: Infected cells death rate in $days^{-1}$
- r: Immune response growth rate in $days^{-1}$
- k: Clearance rate of free virus by immune system
- p: Virus production rate in $TCID_{50}$/ml per day
- c: Virus death rate in $days^{-1}$
- ε: Antiviral efficacy

We estimate key model parameters by fitting the model to clinical trial data and conduct separate analysis for adults and children. For adults, we use the results of the CAPSTONE-1 clinical trial [5] described in [6]; for children, we use the results from the MiniSTONE-2 trial [7] described in [8], as well as viral titer data for children receiving placebo from [9].

When fitting our within-host model to clinical trial results, we solve for the sets of parameter values that produce changes in viral titer levels following treatment that best match data from patients who received baloxavir, oseltamivir, or placebo in clinical trials. Clinical trial publications typically do not report the exact time of day at which viral measurements are taken or when patients first seek treatment after symptom onset. We follow the approach described in [3] to randomly assign values to those time points, as follows. We assume that individuals seek treatment within 48 hours after symptom onset, with the exact timing described by different distributions for adults and children. For adults, we use the gamma distribution reported in [3]; for children, we fit a gamma distribution to the times reported in [10] (Table S1.1). We assume sampling of viral titer occurs uniformly between 6am and 6pm. We assume that the incubation period is fixed and symptom onset occurs 1.97 days after infection [11]. For each candidate set of parameters, we generate patient measurements in three steps: (i) simulate an entire viral titer curve for a single patient using the within-host model with the given parameters, (ii) use the treatment initiation and sampling time parameters just described to simulate random measurement times for the patient, and (iii) calculate the changes in viral titer post treatment at each time point.

To fit the model to clinical data, we first fix the initial conditions and the immune response efficacy (Table S1.2) following results from [12], and then use a simulated annealing algorithm [13] in the SciPy/Python package [14] with 2,000 steps to estimate the remaining parameters, including the efficacy of both antivirals (Table S1.4). The parameter search ranges (Table S1.3) are based on the results reported in [12] and [3] for adults. For children, we initially assume the same parameter ranges as adults, and then expand the ranges of any parameters whose

estimates converge toward a bound. The simulated annealing algorithm seeks to minimize the squared difference between (i) the average changes in the logarithm of viral titer across treated individuals reported from clinical trials and (ii) the corresponding values estimated by our model, summed over the sampling times and the three possible treatments. Due to the large size of the parameter space and the non-linearity of the objective function, we ran the simulated annealing algorithm 1,000 times, starting from different parameter combinations generated using Latin hypercube sampling.

The best set of parameters for adults and children - those minimizing the objective function while producing a sensible viral titer curve - are given in Table S1.4. Figures 1A and 1B show that the model projections with the estimated parameter values are consistent with the clinical trial data. These provide accurate estimates of the average change in viral titer in the days following treatment, but underestimates the variation across patients. Therefore, we introduce additional stochasticity into our simulations of within-host viral trajectories by modeling the duration of the symptomatic period and the peak viral titer as random parameters with specified distributions. For each individual, we draw a random symptomatic period and a random peak viral titer and generate their viral titer curve as follows: (i) throughout the incubation period, the titer is equal to the average titer across all patients, (ii) after symptom onset, the curve maintains the same shape as the average curve but is either compressed or stretched to achieve the given length of their symptomatic period and scaled in magnitude by the same value across all time points to achieve the given height of the peak. The assumed parameter distributions yield sets of simulated patient curves with means and standard deviations that are consistent with the clinical trial data [5,7] (Figure S1.1).

For each simulated patient, we translate their viral titer curve into estimates of daily infectiousness using the following procedure. An individual's total infectiousness on day *d* is given by

$$\text{Infectiousness}_d = \int_{t \in d} log_{10}(V_t) \quad .$$

Then the probability an infectious individual infects one of their susceptible contacts on that day is given by $y_d = \beta \cdot \text{Infectiousness}_d$, where β is a baseline transmission parameter fit to achieve a specified reproduction number (R_0), as follows. We first use the following formula derived from bond percolation theory [15] to estimate the average probability that an infectious individual will transmit the disease to one of their susceptible contacts, *T*:

$$R_0 = T \cdot \left(\frac{< k^2 >}{< k >} - 1 \right)$$

where $<k>$ and $<k^2>$ are the mean degree (i.e., number of contacts) and the mean square degree across all individuals in the population. In estimating *T*, we average across populations that are expected to have different levels of infectiousness, as given by

$$T = \sum_{g \in G} p_g \cdot T_g$$

where *g* indexes over four population subgroups G = {symptomatic adults, asymptomatic adults, symptomatic children, asymptomatic children}, p_g is the proportion of the population in group *g*, and T_g is the probability that an infectious individual from group *g* will transmit the disease to one of their susceptible contacts, and is given by

$$T_g = 1 - (1 - \beta \cdot \overline{\text{Infectiousness}_g})^{\tau_g}$$

Where τ_g is the average duration of the infectious period for group *g* in days and $\overline{\text{Infectiousness}_g} = \frac{\sum_d \text{Infectiousness}_d}{\tau_g}$ is the average daily infectiousness for that group. We can then combine these equations to derive β for a given R_0.

Our model reflects observed differences in peak viral titers for children ($log_{10}(V_{max,c}) = 4.5$ [8]) and adult ($log_{10}(V_{max,a}) = 6.8$ [6]). However, this may stem from differences in the circulating influenza viruses during the seasons in which the trials were conducted, rather than biological differences between children and adults, given that the most significant difference observed between children and adults infected with the same virus is the duration of shedding (longer for children) [16], rather than the magnitude of the peak [17,18]. We therefore adjust the output of the within-host model by scaling the viral titer of children: we take the ratio of the peak viral titers observed between adults and children $\rho = log_{10}(V_{max,a}) / log_{10}(V_{max,c})$, and scale the modeled viral titer of children as $log_{10}(V_{children,scaled}) = \rho \cdot log_{10}(V_{children})$. Table S1.4 provides the assumed distributions for peak viral titer and symptomatic period duration of untreated individuals.

Table S1.1. Parameters for distribution of time from symptom onset to treatment seeking (in days).

Group	Distribution	Source
**Adults**	Γ(shape=4.0, rate=0.2625)	[3]
**Children**	Γ(shape=4.01, rate=0.166)	Fit to [10]

Table S1.2. Fixed parameters and initial conditions for the within-host model.

Parameter	Value	Source
F_0: Initial number of infected cells	0	[12]
U_0: Initial number of susceptible target cells	$4 \cdot 10^8$	[12]
L_0: Initial number of latent cells	0	[4]
k: Immune response effect	1	[12]

Table S1.3. Allowed within-host model parameter ranges for estimation using simulated annealing.

Parameter	Adults	Children	Source
Z_0: Initial immune response	$0.34 \cdot 10^{-3}$ - $0.34 \cdot 10$	$0.34 \cdot 10^{-3}$ - $0.34 \cdot 10$	[3]
V_0: Initial viral titer ($TCID_{50}$/ml)	$0.0077 \cdot 10^{-3}$ - $0.0077 * 10^{4}$	$0.0077 \cdot 10^{-1}$ - $0.0077 \cdot 10^{6}$	[3]
b: Cell infection rate (ml/$TCID_{50}$ per day)	$0.099 \cdot 10^{-3}$ - $0.099 \cdot 10$	$0.099 \cdot 10^{-2}$ - $0.099 \cdot 10^{2}$	[3]
p: Virus production rate ($TCID_{50}$/ml per day)	$1.2 \cdot 10^{-6}$ - $1.2 \cdot 10^{-1}$	$1.2 \cdot 10^{-6}$ - $1.2 \cdot 10^{-1}$	[3]
c: Virus death rate ($days^{-1}$)	$0.081 \cdot 10^{-1}$ - $0.081 \cdot 10^{4}$	$0.081 \cdot 10^{-1}$ - $0.081 \cdot 10^{4}$	[3]
δ: Infected cells death rate ($days^{-1}$)	0.05 - 5.0	0.05 - 5.0	[3]
r: Immune response growth rate ($days^{-1}$)	0.1 - 10	0.1 - 10	[3]
γ: Latent phase rate ($days^{-1}$)	0.01 - 100	0.01 - 100	[12]
$\epsilon_{baloxavir}$: Baloxavir efficacy	0.0 - 1.0	0.0 - 1.0	[3]
$\epsilon_{oseltamivir}$: Oseltamivir efficacy	0.0 - 1.0	0.0 - 1.0	[3]

Table S1.4. Parameter values for the within-host model, estimated by fitting the model to clinical trial data using simulated annealing.

Parameter	Adults (>18)	Children (<18)
Z_0: Initial immune response	0.053	0.081
V_0: Initial viral titer ($TCID_{50}$/ml)	0.0006	78.9
b: Cell infection rate	0.0069	0.0084
p: Virus production rate	0.053	0.0016
c: Virus death rate	0.71	8.2
δ: Infected cells death rate	0.997	0.66
r: Immune response growth rate	1.66	1.28
γ: Latent phase rate	3.1	1.15
$\epsilon_{baloxavir}$: Baloxavir efficacy	0.99998 (95% CI: 0.9993-1.0)	0.9996 (95% CI: 0.9992-1.0)
$\epsilon_{oseltamivir}$: Oseltamivir efficacy	0.9623 (95% CI: 0.9584-0.9702)	0.9668 (95% CI: 0.9429-0.97)
Symptomatic period (days)	*U*(4.7, 6.7)	*U*(5.7, 7.7)
Peak viral titer ($\log_{10}$)	*U*(3.2, 10.6)	*U*(3.1, 10.6)

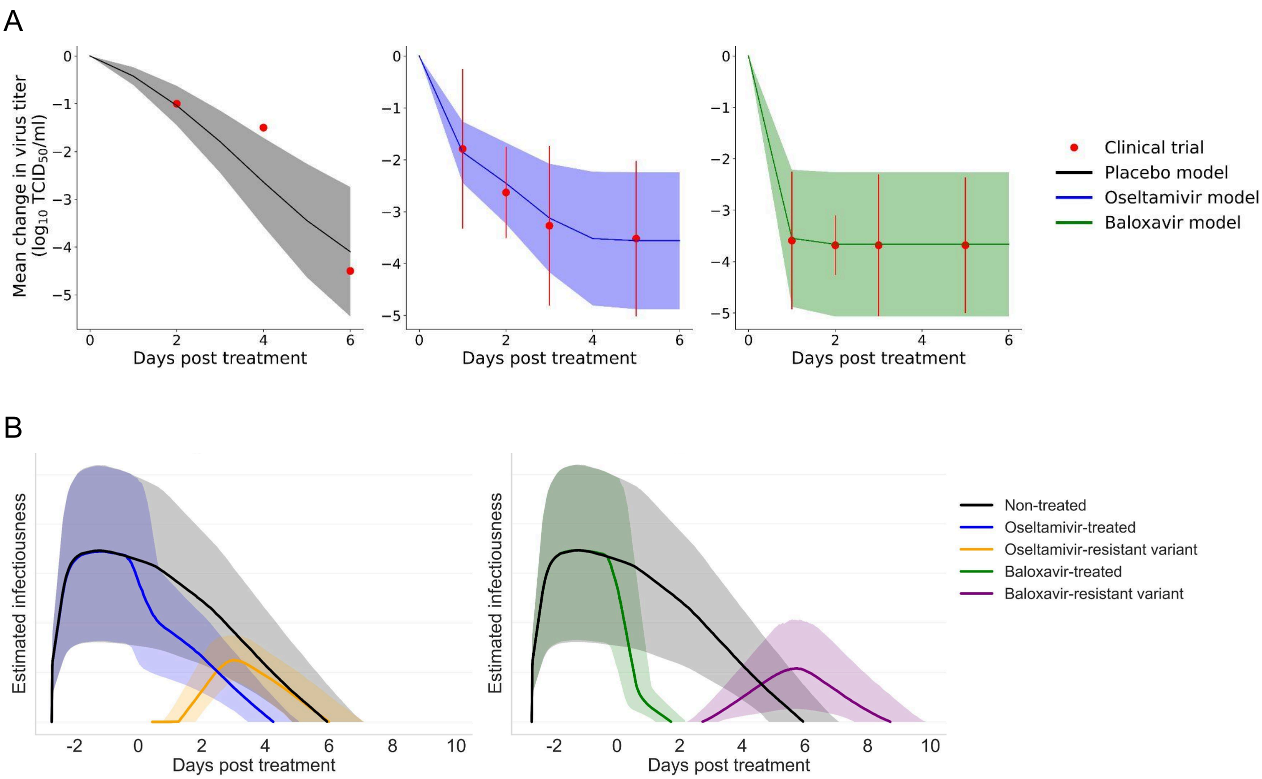

Figure S1.1. Simulated and observed influenza viral titer trajectories, and estimated infectiousness for treated and untreated influenza infections in children. (A) Means and standard deviations of influenza titer trajectories of wild-type virus only across simulated patients (lines and shading) post-treatment in comparison to comparable values reported in clinical trials (dots and error bars) [7,9]. (B) Estimated infectiousness of a group of untreated cases (black line and shading) compared to a corresponding group of treated cases (blue and green lines and shading). For the small fraction of cases who develop antiviral-resistance (Table 1), the orange and purple curves indicate the infectiousness of the resistant virus. The graphs correspond to children treated with oseltamivir on the left, and baloxavir on the right. Lines represent medians and shading represents 95% confidence intervals estimated from 2,000 stochastic simulations. Estimates assume that treatment is initiated between 12 and 24 hours after symptom onset and that infectiousness is logarithmically related to viral load.

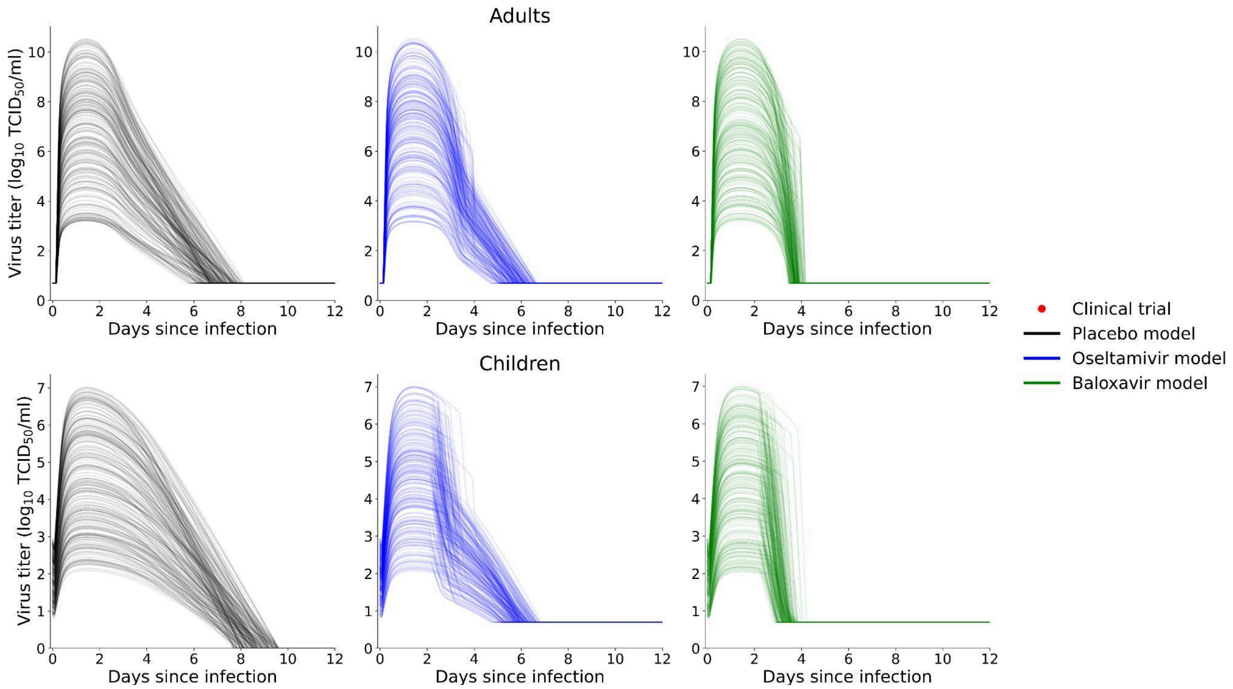

Figure S1.2. Simulated influenza viral titer trajectories for adults and children with and without treatment. Values indicate wild-type virus titer only, assuming that treatment-resistance does not arise. Each graph shows 200 stochastic simulations of our calibrated within host model, in which the duration of the symptomatic period and peak viral titer are random variables. The top and bottom rows correspond to adults and children, respectively.

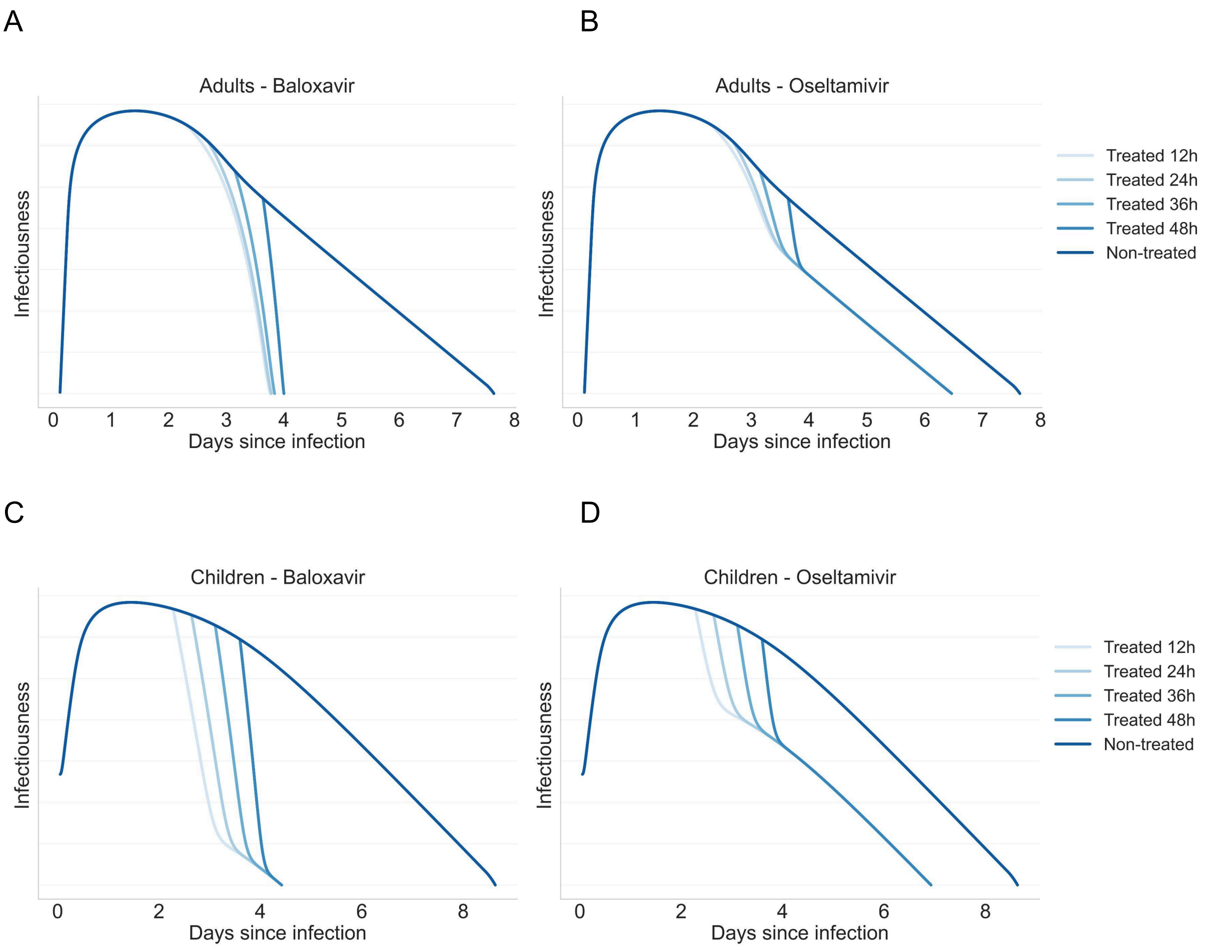

Figure S1.3. Estimated average infectiousness of adults and children treated with either baloxavir or oseltamivir dependent on treatment time following symptom onset. Infectiousness is calculated as the logarithm of model-estimated viral titer. The graphs show estimated averages for infected individuals that are either not treated, or treated within 12 hours, 12-24 hours, 24-36 hours, 36-48 hours of symptom onset, for each of the following combinations: (A) adults receiving baloxavir, (B) adults receiving oseltamivir, (C) children receiving baloxavir, and (D) children receiving oseltamivir.

1.2. Treatment emergent variants

The clinical trial results do not provide detailed viral titer data on the treatment-resistant strains. Therefore, we use other sources to estimate the time at which viral shedding begins, the duration of shedding, and the peak viral titer. For the baloxavir-resistant strains, we used the data provided in [19]. To estimate the probability of baloxavir-resistant variant emergence following combination therapy, we compare the number of cases where treatment resistance emergence is observed during the combination therapy clinical trials [20] with the number we would expect to observe if emergence occurred as often as it did with baloxavir treatment only, per probabilities listed in Table 1. We calculate the probability of emergence with combination therapy to be 57.4% lower than with baloxavir only. We then scale the probability of emergence per age-group and for each virus subtype using this ratio. For oseltamivir-resistant strains, we consider data from a long term study of the emergence of oseltamivir resistance [21]. This study provides patient-level overall viral titer at different multiple time points, along with a variable indicating whether treatment-resistant strains were present at each time point. We assumed that samples positive for treatment-resistant strains were composed equally of the wild-type and the treatment-resistant strains. The resulting parameters used to describe the viral titer trajectory associated with the treatment-resistant variants are given in Table S1.5.

Table S1.5. Parameters for the within-host emergence of treatment-resistant strains.

Antiviral	Parameter	Value	Source
Baloxavir	Start of viral shedding	1 day after initial cessation of shedding of wild-type virus	Refs. [10,19,22,23]
	Shedding duration (days)	$U(5.0, 7.0)$	
	Peak viral titer ($\log_{10}$)	Adults: $U(0.3,\ 4.3)$<br>Children: $U(0.5,\ 4.5)$	
Oseltamivir	Start of viral shedding (days post onset of symptoms)	0.404 × symptomatic period duration	Ref. [21]
	Shedding duration (days)	Adults: $U(3.27,\ 5.27)$<br>Children: $U(3.71,\ 5.71)$	
	Peak viral titer ($\log_{10}$)	Adults: $U(2.53,\ 4.23)$<br>Children: $U(1.94,\ 3.76)$	

2. Influenza transmission model

To model the spread of a wild-type influenza virus, oseltamivir-resistant influenza virus, and baloxavir-resistant influenza virus, we use an individual-based model of influenza transmission with a realistic network structure that captures contact patterns with households, schools, workplaces and other points of interest. The model tracks the infection status of each individual and the infectiousness of an infected individual varies through time according to the within-host viral dynamic model described in Section 1.

2.1 Population and network structure

A contact network with 10,000 individuals that reflects the typical structure of a United States community is constructed as follows. Each individual is assigned to a household and an age group (0-4y, 5-17y, 18-49y, 50-64y, or 65+ years) based on United States Census data (Public Use Microdata Sample) [24]; all individuals living in the same household are connected to each other.

Individuals are assigned to schools according to data reflecting the distribution of school sizes, enrollment per age, and staff in the United States [25–28] and assigned to workplaces based on age-specific labor participation rates, unemployment rates, and business sizes [29–31]. We then create contacts between people assigned to the same school or workplace as well as contacts in the community at large based on the venue-specific contact rates for the United States estimated in [32]. Specifically, for a given individual and a given venue (i.e., school, work or community), we determine their number of contacts in that venue by drawing a random value from a Poisson distribution with mean equal to that reported contact rate (Tables S2.1-S2.4). We do not consider any infection-induced changes in individual behavior, such as absenteeism, or community interventions, such as school closures.

The contact matrices used are given in Tables S2.1 to S2.4, where the contact matrix at home is calculated by averaging over 100 randomly generated networks, while the contact matrices for the remaining locations correspond to the ones reported in ref. [32], aggregated for the five age groups modeled.

On weekdays, individuals have contacts with other individuals in their home, community, work, and school networks. On weekends, contacts are restricted to the home and community networks.

To be able to compare interventions and save processing time, networks are reused for 10 simulations and are the same across scenarios. When running 500 simulations we therefore only need to generate 50 different networks, independently of the number of scenarios run.

Finally, we determine which individuals are at high risk of severe outcomes upon infection. The proportion of high-risk individuals within each age group is determined using a previously published methodology [33] and is shown in Table S2.5. For each individual, we randomly assign them to the high-risk group using their age group's proportion.

Table S2.1. Home contact matrix. Average number of daily contacts by age group at home. Calculated by averaging the number of contacts at home across 100 networks.

		Individual age group (years)				
		**0-4**	**5-17**	**18-49**	**50-64**	**65+**
**Individual age group (years)**	**0-4**	0.6	0.8	2.5	0.2	0.1
	**5-17**	0.3	1.1	2.2	0.4	0.1
	**18-49**	0.4	0.9	1.9	0.5	0.1
	**50-64**	0.1	0.4	1	1.6	0.4
	**65+**	0	0.1	0.5	0.6	1.8

Table S2.2. School contact matrix. Average number of daily contacts by age group at school.

		Individual age group (years)				
		**0-4**	**5-17**	**18-49**	**50-64**	**65+**
**Individual age group (years)**	**0-4**	0.7	1.3	0.2	0.1	0.0
	**5-17**	0.5	8.3	1.7	0.3	0.0
	**18-49**	0.0	0.7	1.3	0.1	0.0
	**50-64**	0.0	0.3	0.2	0.0	0.0
	**65+**	0.0	0.1	0.1	0.0	0.0

Table S2.3. Work contact matrix. Average number of daily contacts by age group at work.

		Individual age group (years)				
		**0-4**	**5-17**	**18-49**	**50-64**	**65+**
**Individual age group (years)**	**0-4**	0	0	0	0	0
	**5-17**	0	0.0	0.1	0.1	0.0
	**18-49**	0	0.1	3.2	1.3	0.1
	**50-64**	0	0.1	3.0	1.3	0.1
	**65+**	0	0.0	0.4	0.2	0.0

Table S2.4. Community contact matrix. Average number of daily contacts by age group at other locations.

		Individual age group (years)				
		**0-4**	**5-17**	**18-49**	**50-64**	**65+**
**Individual age group (years)**	**0-4**	0.2	0.5	1.2	0.5	0.4
	**5-17**	0.2	0.5	1.2	0.5	0.4
	**18-49**	0.2	0.5	1.2	0.5	0.4
	**50-64**	0.2	0.5	1.2	0.5	0.4
	**65+**	0.2	0.5	1.2	0.5	0.4

2.2 Transmission model parameters

The model tracks the disease status of each individual throughout the course of the influenza season. Upon infection, individuals enter an incubation period (with log-normally-distributed duration [11]) before progressing to either a symptomatic or asymptomatic [34] infectious period (with uniformly-distributed durations [6,8]). Symptomatic cases may be hospitalized and then either recover or die (at age-specific hospitalization and mortality rates [35]). We assume that antiviral-resistant infections have the same hospitalization and mortality rates as antiviral-sensitive infections, if untreated. Recovered individuals enjoy permanent cross-immunity to all viruses for the duration of the simulation.

Once infected, the viral titer of an individual follows the trajectory described by the output from the within-host model, adjusting the curve as necessary based on the individual's exact incubation period, symptomatic period, and peak viral titer. Their infectiousness is then set equal to the base 10 logarithm of their viral titer [16]. The probability of infecting a contact is set to the reproduction number of the circulating virus scaled by the individual's infectiousness at that point in time.

To keep the computational cost of the simulation model tractable, we model one time step per day, where the contacts that occur depend on whether the day is a weekday or weekend (details in Table S2.5). The infectiousness of an individual for that time step is then calculated as their average infectiousness from the beginning to the end of that day, set as 8am to 10pm, using their viral titer curve. Then the probability that this individual infects one of their contacts is calculated by multiplying their infectiousness for that day by the average probability of infection during a contact.

The parameters used to describe a new infection with one of the treatment emergent variants are the same as that used for the wild-type strain. Therefore the incubation period, symptomatic period, and viral titer curves are the same. The probability of infection during a contact for the variants is then calibrated so that the variants have a reproductive number set to the selected input, therefore any loss in fitness incurred by a treatment-resistant variant is reflected in that transmission rate and not in the viral titer of infected individuals.

We then assume that antivirals have no effect at all on their respective treatment-resistant variant, while using oseltamivir to treat an individual infected with the baloxavir-resistant variant has the same effect as treating an individual infected with the wild type virus, and similarly using baloxavir to treat an infection with the oseltamivir-resistant variant works normally. We assume that no treatment emergence can occur from treating an individual infected from a treatment-resistant variant.

We assume any infection provides cross-immunity to all other variants, so that each individual may only be infected once per simulation.

Table S2.5. Transmission model parameters. Values given as five-element vectors are age-stratified with values corresponding to 0-4, 5-17, 18-49, 50-64, 65+ year age groups, respectively.

Parameter	Value	Source
Population size	10,000	
Initial number of infections	5 low-risk 18-49y individuals<br>5 low-risk 50-64y individuals	
Proportion of symptomatic individuals treated	Scenario specific	
Distribution of time to treatment from symptom onset in hours, [0-12, 12-24, 24-36, 36-48] ranges respectively	Children: [52.2%, 32.8%, 13.4%, 1.6%]<br>Adults: [13%, 40%, 29%, 18%]	Hirotsu et al. 2020; Du et al. 2020 [3,10]
High-risk proportion, age specific (%)	[8.4, 14.3, 18.9, 33.0, 47.1]	Methodology detailed in Pasco et al. [33]
Basic reproductive number R_0	Wild-type: 1.28 for seasonal scenarios, and 2.5 for pandemic scenarios<br>Variants: scenario specific	Biggerstaff et al. [36]
Average transmission probability per contact	Fitted to R_0	
Incubation period (in days)	Lognormal (0.6575,0.2009)	Lessler et al. [11]
Asymptomatic rate, age specific (%)	[18.9, 18.9, 25.3, 25.3, 25.3]	Ip et al. [34]
Relative infectiousness of asymptomatic individuals compared to symptomatic	0.86	Ip et al. [34]
Relative duration of viral shedding of asymptomatic individuals compared to symptomatic	0.8	Loeb et al. [37]
Contact patterns	Section S2.1, Tables S2.1-2.4	Mistry et al. [32]

3. Hospitalizations, deaths, and DALYs calculations

We calculate the expected number of hospitalizations and deaths occurring in a given simulation once a simulation is over, we do not model those at each simulation point. They are calculated by multiplying the number of infected individuals by their respective age and risk group's probability of hospitalization and death, including any reduction in those outcomes if treated, using the parameters given in Table S3.1.

Because estimates of the probability of hospitalization and death after infection vary greatly in the literature, we used reports of total influenza burden in the United States [35] - symptomatic illnesses, hospitalizations, and deaths - to calculate those probabilities. For each of the five age groups modeled, and each season from 2010-2011 to 2018-2019, we calculated the hospitalization rate of symptomatic individuals and the death rate of hospitalized individuals and used the average across all seasons as input to our calculations.

Table S3.1. Probability of severe outcomes for high- and low-risk individuals and impact of antivirals on severe outcomes. Values given as five-element vectors are age-stratified with values corresponding to 0-4, 5-17, 18-49, 50-64, 65+ year age groups, respectively.

Parameter	Value	Source
Relative risk of hospitalization for high-risk individuals compared to low-risk in their age group	3	Hanslik et al. [38]
Relative risk of death for high-risk individuals compared to low-risk in their age group	3	Van Kerkhove et al. [39]
Symptomatic case hospitalization rate (%)	Overall: [0.70, 0.27, 0.56, 1.06, 9.09]<br>Low-risk: [0.60, 0.21, 0.41, 0.64, 4.68]<br>High-risk: [1.79, 0.64, 1.22, 1.92, 14.04]	Estimated from CDC data [35]
Hospitalized fatality ratio (%)	Overall: [0.98, 1.66, 4.24, 6.74, 11.90]<br>Low-risk: [0.84, 1.29, 3.08, 4.06, 6.13]<br>High-risk: [2.51, 3.88, 9.23, 12.18, 18.38]	Estimated from CDC data [35]
Relative risk of hospitalization of treated individuals	Oseltamivir, low-risk: 0.75<br>Oseltamivir, high-risk: 0.75<br>Baloxavir, low-risk: 0.41<br>Baloxavir, high-risk: 0.24	Hsu et al. [40], Neuberger et al. [41]
Relative risk of death given hospitalization of treated individuals	Oseltamivir, low-risk: 0.5<br>Oseltamivir, high-risk: 0.25<br>Baloxavir, low-risk: 0.5<br>Baloxavir, high-risk: 0.25	Hsu et al. [40], Neuberger et al. [41]

For each scenario we calculate the Disability-adjusted life years (DALYs) lost. DALY is a metric that captures the overall burden of a disease, by capturing (i) years lived with disability, either due to some temporary loss of quality of life while symptomatic or hospitalized, or some permanent disability due to infection or hospitalization, and (ii) the years of life lost due to premature death [42].

Once we have calculated the number of hospitalizations and deaths per age group, we can calculate the DALYs lost following the methodology described in [43]. In short, DALYs are the sum of years of life lost (YLL) and years lived with disability (YLD).

YLL represents premature deaths and is calculated by multiplying the number of deaths in an age group by the average remaining life expectancy, which can be found in [44].

YLD represents a decrease in quality of life due to a temporary infection or hospitalization, or some permanent condition following a severe infection. Sah et al. [43] consider the six following outcomes in the calculation of YLD: uncomplicated outpatient cases; uncomplicated hospitalized cases; otitis media in complicated outpatient cases; acute otitis media leading to long-term hearing impairment in complicated outpatient cases; acute respiratory distress syndrome (ARDS) in complicated hospitalizations; and pneumonia in complicated hospitalized cases. Each has a different probability, duration, and associated disability weight. Summing over all those we obtain total YLD.

4. Additional results

4.1 Infectiousness

Table S4.1 uses the average viral titer curves shown in Figure S1.2 to calculate the relative reduction in total infectiousness of individuals treated with each antiviral for different treatment timing. Total infectiousness is calculated as the total area under the infectiousness curve from infection to end of shedding, as given by $\text{Infectiousness} = \int_t log_{10}(V_t)$. The reduction in infectiousness calculated closely match the observed reduction in household attack rates when treating index patients with baloxavir during the CENTERSTONE trial [45].

Table S4.1. Average reduction in total infectiousness (over the course of the entire infectious period) resulting from treatment with an antiviral. The reduction depends on the antiviral used, the age group of the individual treated, and the time at which treatment is initiated after symptom onset. Values are estimated using our within-host viral dynamic model assuming the average incubation period, symptomatic period, and peak viral titer. Infectiousness is calculated as the logarithm of viral titer. Combination therapy has the same effect as baloxavir when treating an infection by a baloxavir-susceptible virus.

	**Reduction in infectiousness under antiviral treatment (%)**			
	**Adults**		**Children**	
**Treatment timing (hours after symptom onset)**	**Baloxavir**	**Oseltamivir**	**Baloxavir**	**Oseltamivir**
0-12	35	19	53	31
12-24	34	18	49	29
24-36	32	17	43	26
36-48	28	15	38	24
**0-48**	**32**	**17**	**46**	**27**

4.2 Impact of expanded antiviral treatment on influenza-related infections, hospitalizations and deaths during A(H1N1) seasons, A(H3N2) seasons, and hypothetical pandemics.

Figures 2A and 2B in the main text project the impact of antiviral strategies on deaths per 10,000 during a typical influenza A(H3N2) season and a hypothetical A(H3N2) influenza pandemic. Figure S4.1 provides the corresponding estimates of projected infections (as a percent of the population) and hospitalizations (per 10,000). Figure S4.2 provides a full set of estimates (infections, hospitalizations, and deaths) for a typical A(H1N1) season and hypothetical A(H1N1) pandemic.

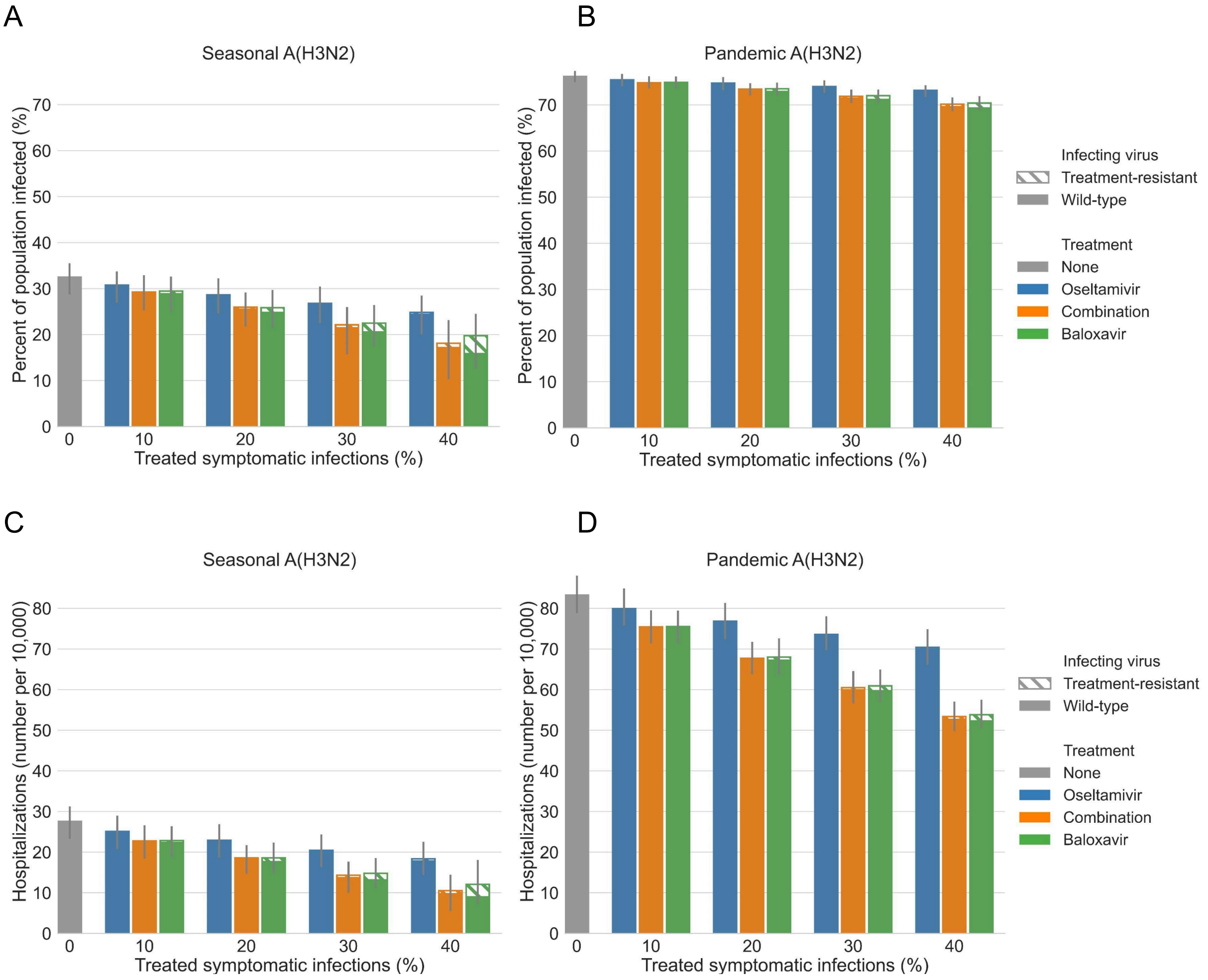

Figure S4.1. Projected impact of expanding treatment with oseltamivir, baloxavir or combination therapy for seasonal and pandemic influenza A(H3N2). For (A) a seasonal virus with wild-type R_0=1.28 and treatment-resistant R_0=1.15 and (B) a pandemic virus with wild-type R_0=2.5 and treatment-resistant R_0=2.25, bar heights indicate the median percent of the population infected with a wild-type (solid bar) or treatment-resistant (hatched bar) virus, based on 500 stochastic simulations. Gray lines indicate 95% confidence intervals. The x-axes indicate the proportion of symptomatic cases that receive treatment; colors correspond to the type of treatment administered. (C) and (D) provide corresponding estimates for influenza-related hospitalizations per 10,000.

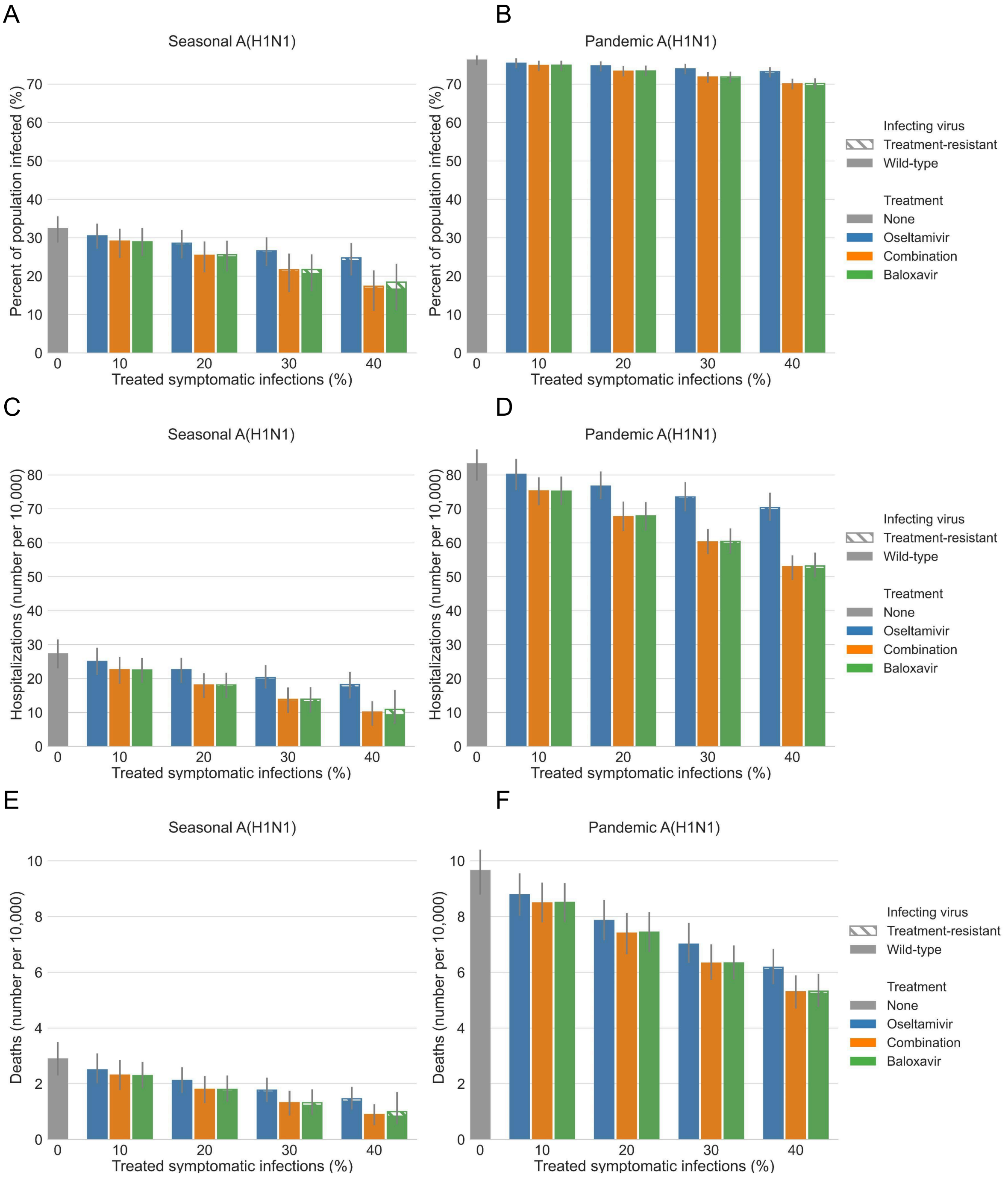

Figure S4.2. Projected impact of expanding treatment with oseltamivir, baloxavir or combination therapy for seasonal and pandemic influenza A(H1N1). For (A) a seasonal virus with wild-type R_0=1.28 and treatment-resistant R_0=1.15 and (B) a pandemic virus with wild-type R_0=2.5 and treatment-resistant R_0=2.25, bar heights indicate the median percent of the population infected with a wild-type (solid bar) or treatment-resistant (hatched bar) virus, based on 500 stochastic simulations. Gray lines indicate 95% confidence intervals. The x-axes indicate the proportion of symptomatic cases that receive treatment; colors correspond to the type of treatment administered. (C) and (D) provide corresponding estimates for hospitalizations per 10,000; (E) and (F) provide corresponding estimates for deaths per 10,000.

4.3 Attack rate, hospitalizations, and deaths based on groups treated

Figure S4.3 expands on the results shown in Figures 1A and 1B. We show the impact of allocating one antiviral treatment (oseltamivir, combination therapy, or baloxavir) to high-risk individuals, adults (18y+), children (0-17y), or all individuals, with 20% of symptomatic individuals in the selected group receiving treatment. From this we can see that treating children only can reduce transmission of influenza to a greater extent than when treating the other groups, however treatment-resistant emergence is also more likely to occur in that case.

Looking at severe outcomes, hospitalizations and deaths, we see that treating high-risk individuals or adults only is as effective as treating children only, and even more effective when focusing on deaths despite the higher cumulative attack rate in those scenarios.

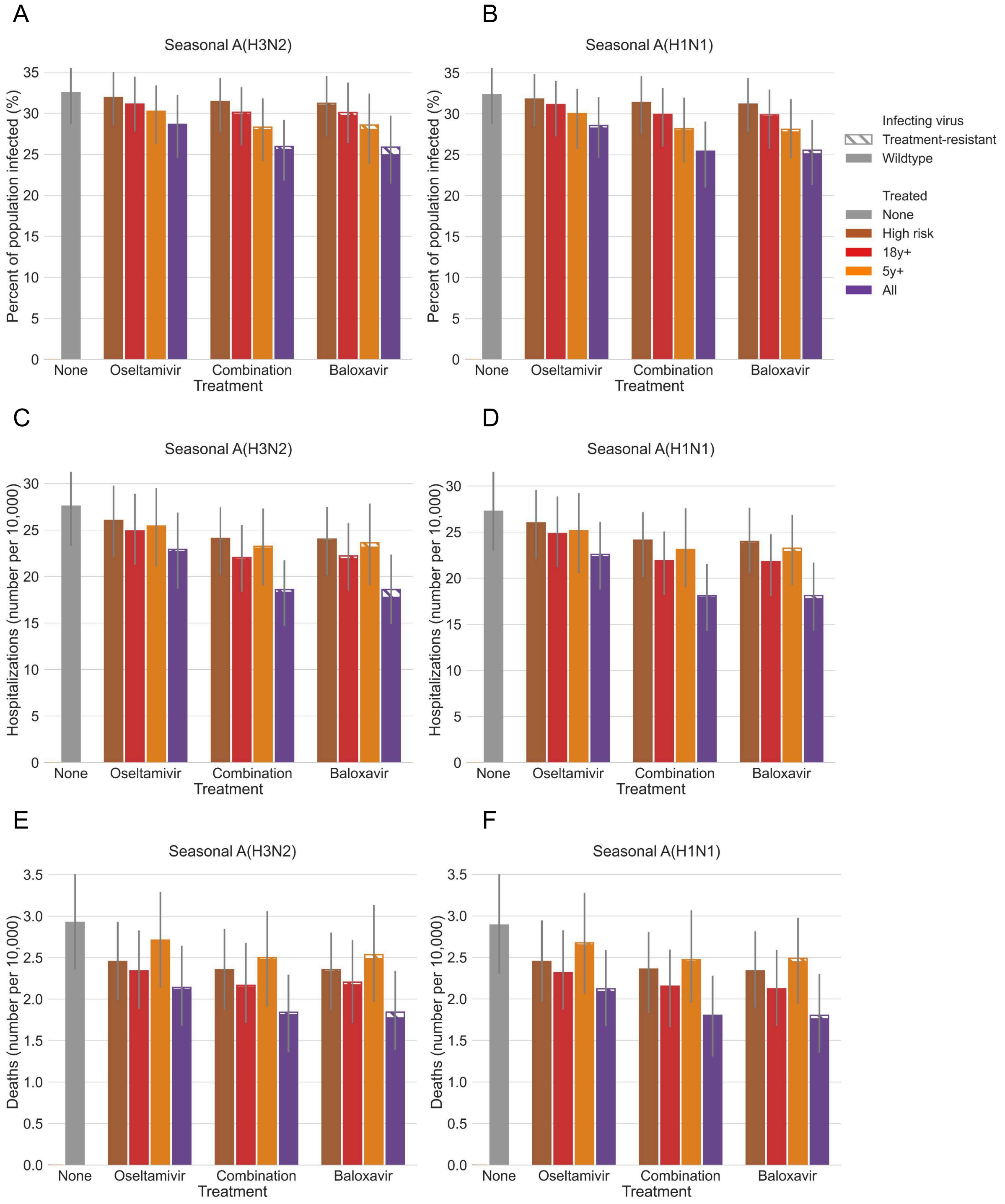

Figure S4.3. Impact of age-restricted treatment on the proportion of the population infected, hospitalized, or dying with wild-type influenza (solid) and treatment-resistant (hatched) influenza viruses. For (A,C,E) A(H3N2)-dominant seasons or (B,D,F) A(H1N1)-dominant seasons. Simulations assume reproduction numbers (R_0) of 1.28 for the wild-type virus and 1.15 for the baloxavir-resistant virus. Bar heights and gray lines indicate medians and 95% confidence intervals across 500 simulations.

4.4 Share of infections due to treatment-resistant variants

Figure S4.4 provides estimates for the proportion of infections caused by treatment-resistant viruses under different assumptions for the fitness of the treatment-resistant strains. Oseltamivir-resistant variants never represent a significant portion of total cases in the scenarios considered. Baloxavir-resistant strains are expected to reach larger proportions during influenza A(H3N2) seasons than A(H1N1) seasons, while the opposite holds for oseltamivir.

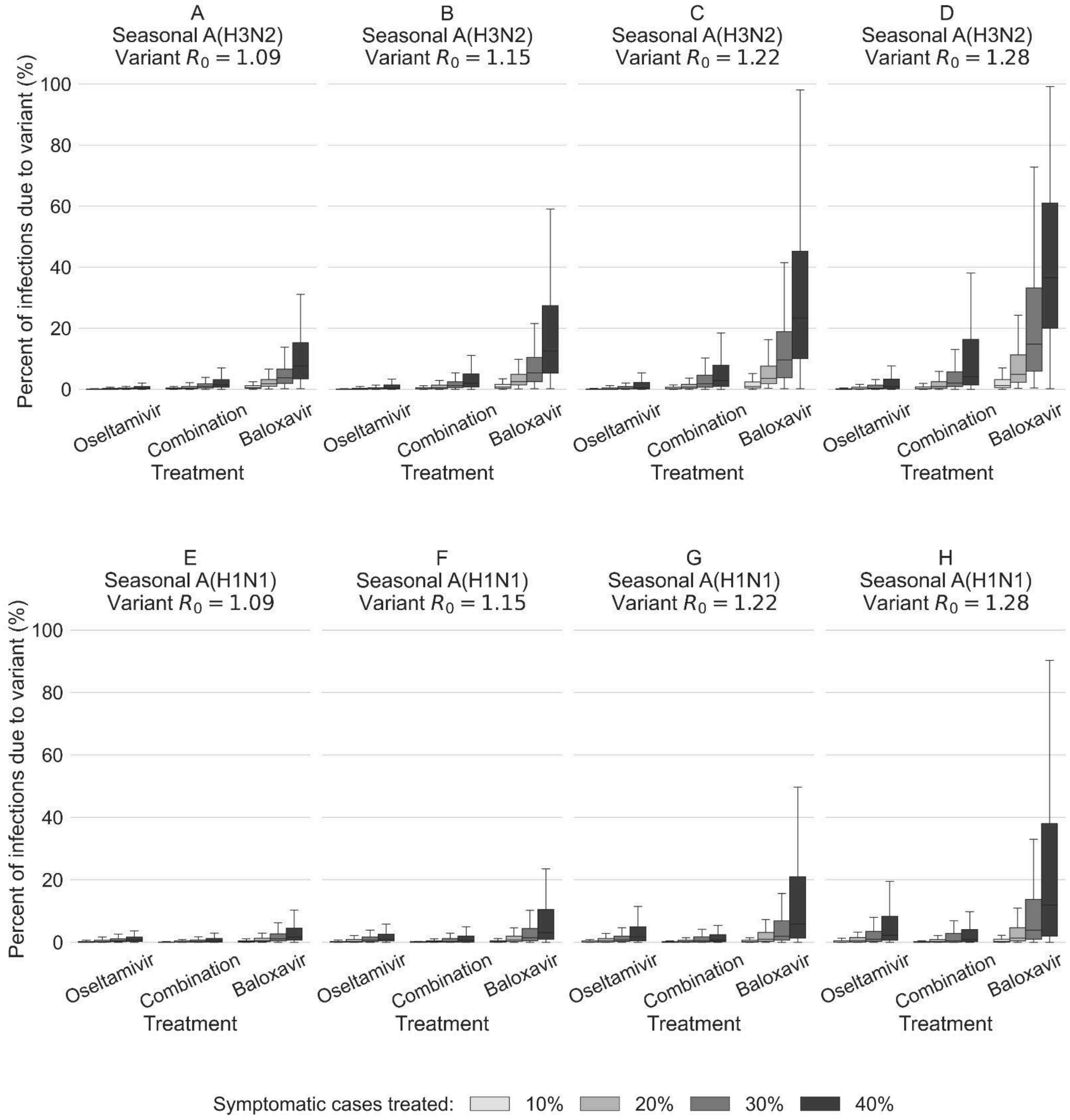

Figure S4.4. Impact of treatment on the proportion of all infections due to treatment-resistant strains depending on the fitness of the treatment-resistant strain. For (A, B, C, D) A(H3N2)-dominated seasons or (E, F, G, H) A(H1N1)-dominated seasons. Combination therapy leads to baloxavir-resistant variant emergence only. The x-axes indicate the type of treatment administered; colors correspond to the proportion of symptomatic cases that receive treatment. All scenarios assume a wild-type R_0 of 1.28. The treatment-resistant R_0 differs as follows: (A, E) 85% of wild-type (1.09); (B, F) 90% of wild-type (1.15); (C, G) 95% of wild-type (1.22); (D, H) equal to wild-type (1.28).

4.5 Trigger levels

In the results that follow, we assume a wild-type R_0 of 1.28 and treatment-resistant R_0 of 1.15. We analyze the impact of using thresholds on observed incidence from treatment-resistant variants to trigger a change in antiviral allocation. Allocation of a treatment is stopped altogether for the season when the proportion of total weekly new cases due to the corresponding antiviral exceeds a chosen trigger level. We show how such triggers can prevent treatment-resistant variants from becoming dominant. In Figure S4.5, we show the probability that a variant's weekly incidence ever represents 20% or more of total new cases at any point in a given season as different groups receive a given antiviral (different colors) and as the trigger level changes (x-axis).

We see that the probability that a variant's incidence ever exceeds 20% of total new weekly cases strongly depends on the variant considered and the influenza strain circulating. In general, treating more people leads to a higher likelihood of exceeding that limit, with the exception of treating either everyone or only 5y+ with baloxavir. In that case, not treating children aged 0y to 4y has very little effect, as the probability of emergence is high in all age groups (Table 1). But in all cases, a lower trigger level reduces the likelihood of the variant's incidence exceeding 20% of total new cases, so that if one wanted to keep that probability under a certain limit they could determine the necessary trigger level. For instance, if aiming for a 10-20 rule - keeping under 10% the probability of variant incidence exceeding 20% of new cases - triggers should be in place at 2%, 2%, and 16% of weekly incidence when influenza A(H3N2) is circulating and baloxavir is used to treat everyone, 5y+, and adults respectively (Figure S4.5A); if influenza A(H1N1) is circulating the triggers should be at 4% and 6% when treating everyone and 5y+ respectively. When using combination therapy (Figure S4.5C) and influenza A(H3N2) is circulating, thresholds should be set at 7% and 9% when treating everyone and 5y+ respectively. In all other scenarios considered, a trigger is not necessary to satisfy this rule. In particular, under the set of parameters considered, the oseltamivir-resistant variant (Figure S4.5B) is not expected to exceed 20% of total incidence with probability greater than 10%.

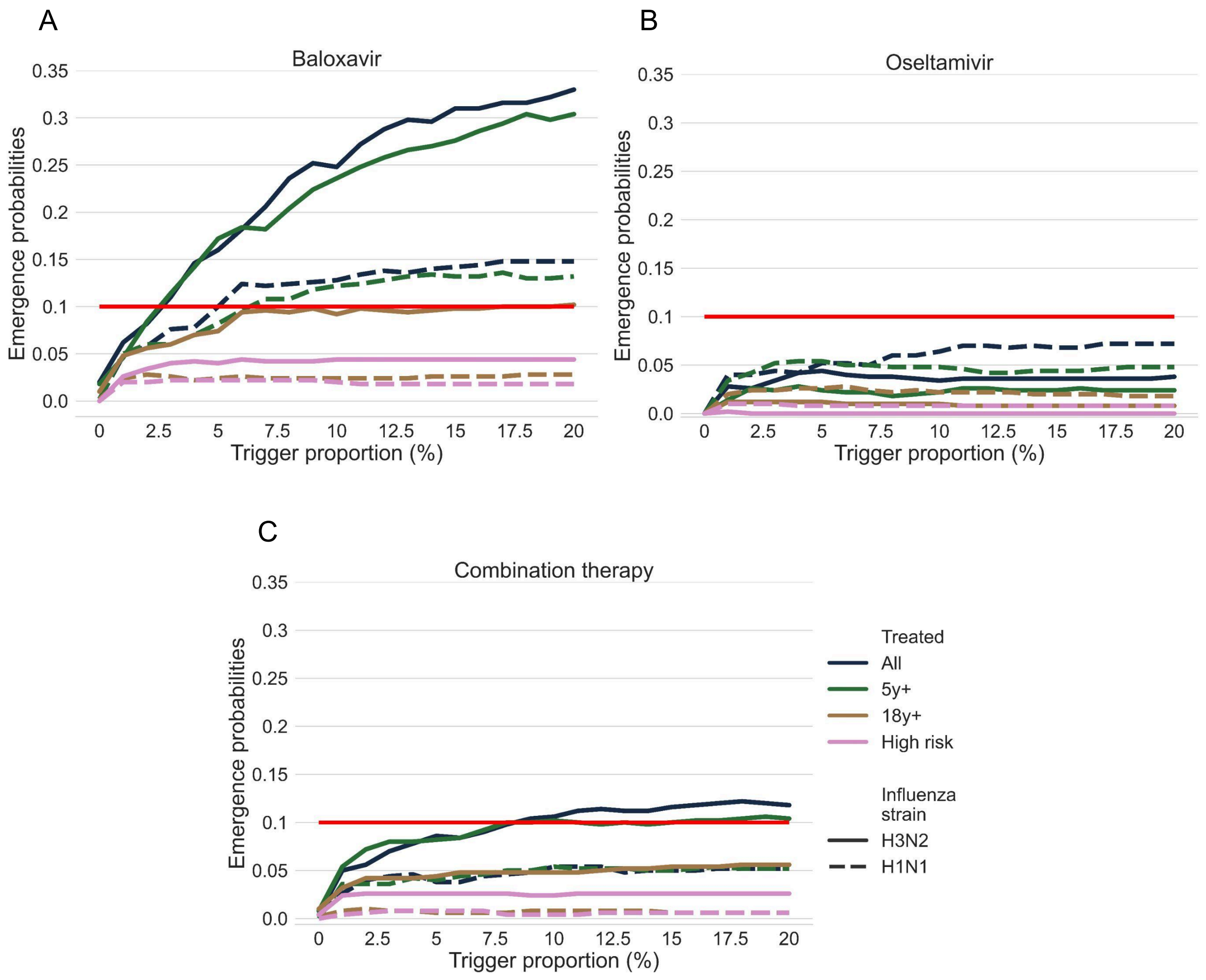

Figure S4.5. Probability of antiviral-resistant strains ever exceeding 20% of total weekly influenza incidence depending on the trigger for suspending treatment. This assumes that 20% of symptomatic cases in the specified group are treated with either (A) baloxavir, (B) oseltamivir, or (C) combination therapy. The x-axes indicate the relative frequency of resistant infections at which treatment is suspended for the remainder of the season. The graphs depict results for both A(H3N2)-dominated (solid lines) and A(H1N1)-dominated (dashed lines) seasons, assuming a wild-type R_0=1.28 and treatment-resistant R_0=1.15. Probabilities are calculated as the proportion of simulations in which weekly incidence reaches 20% or more of total, across 500 simulations. The red solid line corresponds to a 10% chance that antiviral resistance will surpass 20% relative incidence sometime during the influenza season.

4.6 Comparing DALYs lost to risks of oseltamivir resistance

Whereas Figures 3A and 3B in the main text compare DALYs lost to risks of baloxavir resistance, Figure S4.6 compares DALYs lost to the risk that oseltamivir resistance will exceed 20% relative incidence. Rather than a trade-off, we find that the strategies that reduce DALYs lost also reduce resistance risks. However, the best strategies are generally those that expand the use of baloxavir while limiting the use of oseltamivir, and thus are associated with the largest risks of baloxavir resistance (Figure 3).

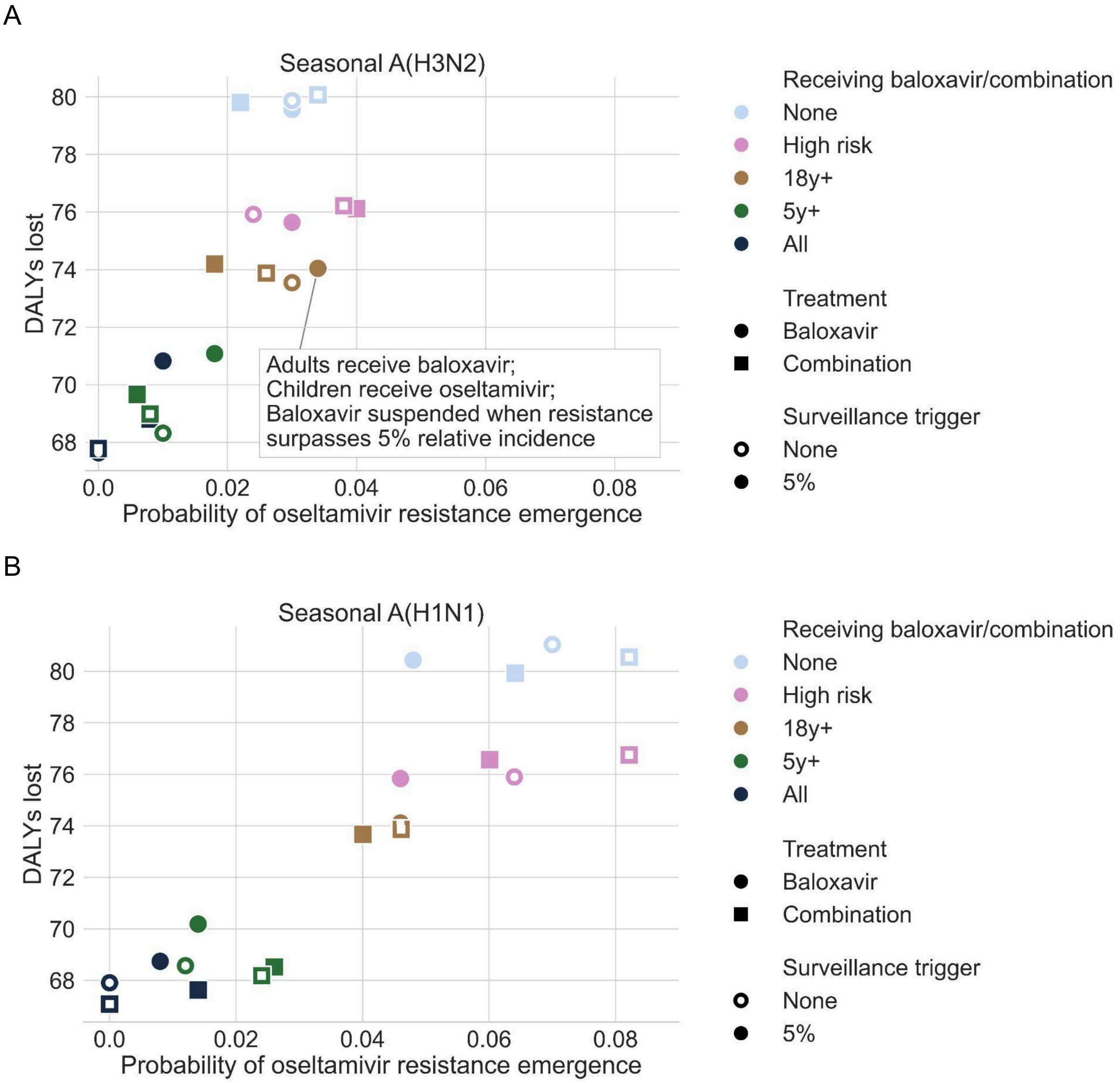

Figure S4.6.Trade-off between public health benefits of antiviral treatment (DALYs lost per 10,000 individuals) and the risk that oseltamivir-resistant viruses will emerge and spread. Emergence is defined as the probability of exceeding 20% of incident infections anytime during the season. For (A) influenza A(H3N2)-dominant seasons and (B) influenza A(H1N1)-dominant seasons. Each of the 20 antiviral treatment strategies assumes that 20% of symptomatic cases receive treatment and specifies the following treated subpopulations to receive either baloxavir (circles) or combination therapy (squares) rather than oseltamivir: none (light blue), high risk individuals only (pink), adults over 18y (brown), children and adults over 5y (green), or all (black). Each strategy also either does (filled symbols) or does not (open symbols) include a surveillance-based trigger for suspending baloxavir and combination therapy when baloxavir-resistant viruses surpass 5% relative incidence, and for suspending oseltamivir when oseltamivir-resistant viruses surpass 5% relative incidence. The estimates are medians across 500 stochastic simulations, and assume that the basic reproduction number (R_0) of baloxavir-resistant variants is 10% lower than that of the wild-type.

4.7 Timing of emergence and subsequent circulation

Figure S4.7 shows the estimated timing of emergence of the baloxavir-resistant variant when baloxavir is used to treat different groups and baloxavir treatment is suspended when resistance reaches 5% of weekly incidence. Specifically, it shows the first week during which the variant represents 20% or more of the proportion of new total weekly cases. To put it in the context of the epidemic's progression we express this relative to the week during which the epidemic peaks. For instance, if in a given simulation the epidemic peaks on week 8 and the variant's incidence exceeds 20% of total on week 10, then the variant's weekly incidence exceeds 20% of total 2 weeks after peak week.

Figure S4.7 also provides two sample simulations where influenza A(H3N2) is circulating and 20% of all symptomatic individuals are treated with baloxavir. The left panels show weekly incidence, splitting cases due to the wild-type virus, the treatment-resistant variant, and total, while the right panels represent the proportion of weekly incidence due to the variant. In both panels the vertical dotted red line represents the first week during which the treatment-resistant variant represents 20% or more of new weekly cases. In the first example, baloxavir resistance surpasses 20% of weekly incidence 8 weeks after peak, with the resistant strain representing a small portion of all infections throughout the season but the only virus remaining at the end of the season. In the second example, resistance reaches the 20% threshold five weeks before peak but disappears before the end of the season.

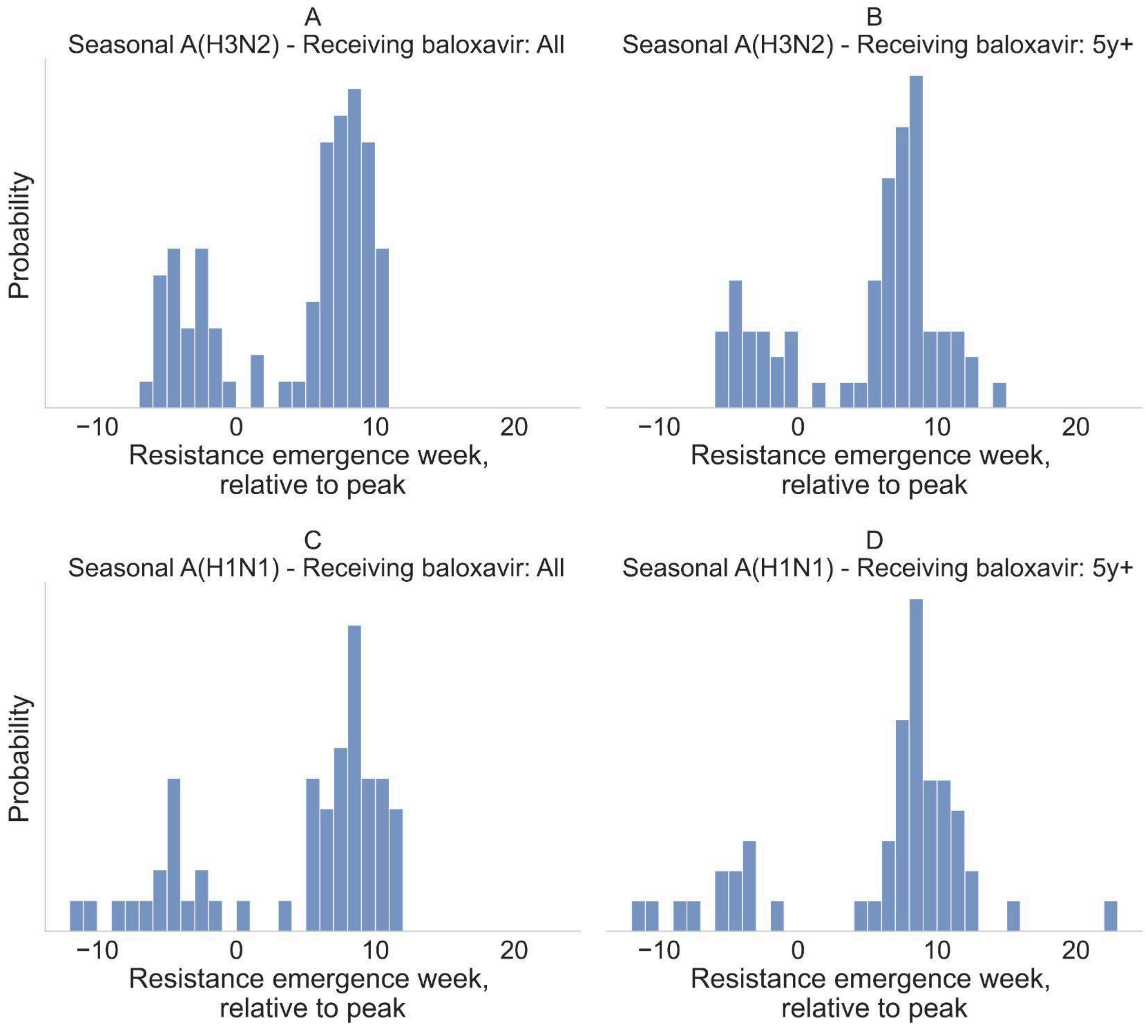
A
Seasonal A(H3N2) - Receiving baloxavir: All
B
Seasonal A(H3N2) - Receiving baloxavir: 5y+
C
Seasonal A(H1N1) - Receiving baloxavir: All
D
Seasonal A(H1N1) - Receiving baloxavir: 5y+
Probability
Probability
−10
0
10
20
Resistance emergence week,
relative to peak

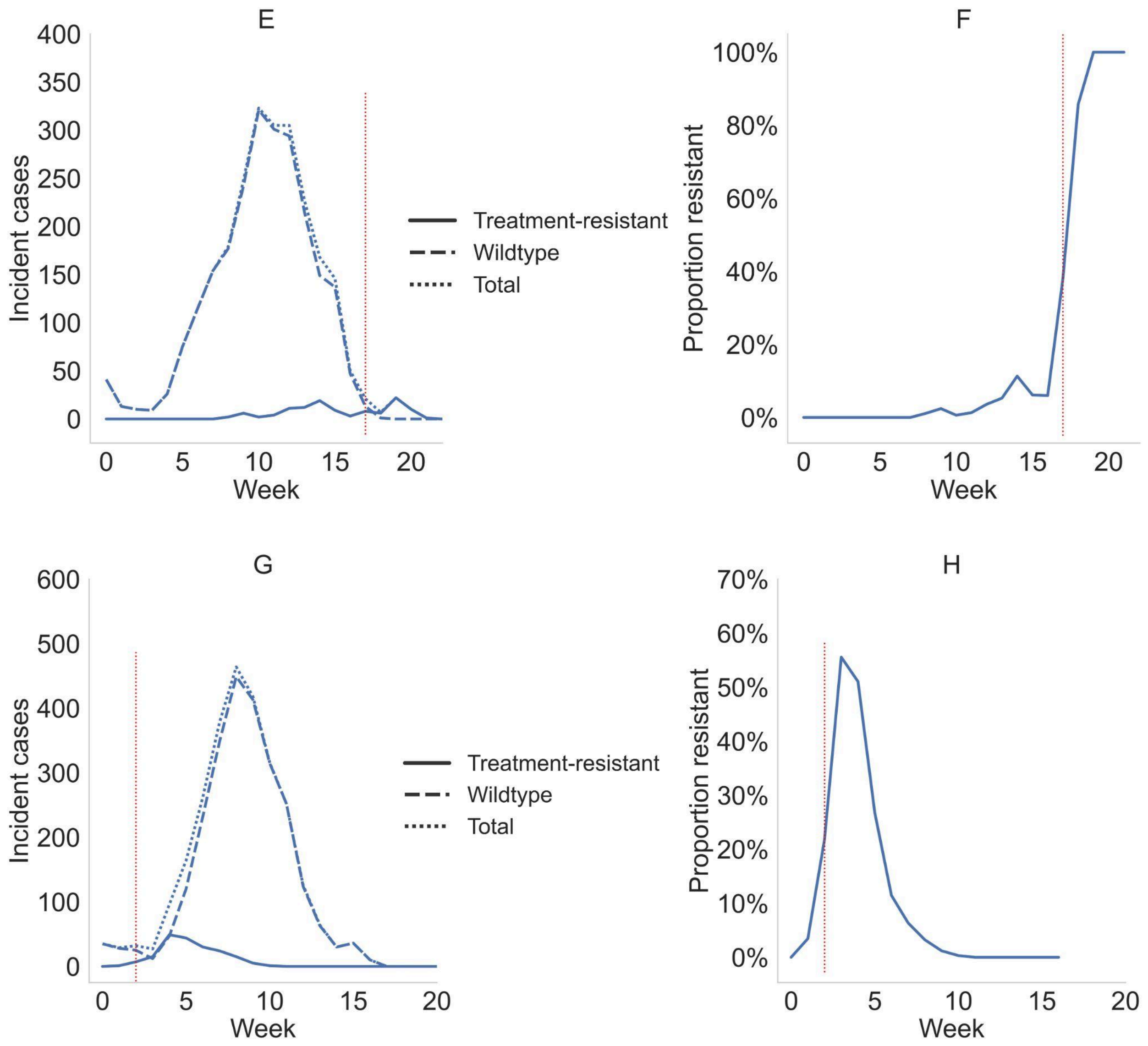

Figure S4.7. Timing of baloxavir-resistant emergence assuming that 20% of symptomatic cases are treated and that baloxavir treatment is suspended when resistance reaches 5% of relative incidence, incidence first exceeding 20% of total with sample simulations. All scenarios assume wild-type R_0=1.28 and treatment-resistant R_0=1.15. The top four graphs show distributions of the first week where baloxavir-treatment resistant infections exceed 20% of incidence, assuming (A) A(H3N2) is circulating and all treated patients receive baloxavir, (B) A(H3N2) is circulating and only treated patients over age 5 receive baloxavir (younger patients receive oseltamivir), (C) A(H1N1) is circulating and all treated patients receive baloxavir, (D) A(H1N1) is circulating and only treated patients over age 5 receive baloxavir. The remaining panels provide results for two example simulations assuming that A(H3N2) is circulating and 20% of symptomatic cases receive baloxavir. (E, G) Weekly incidence of overall, wild-type and treatment resistant infections. (F, H) Proportion of new infections that are treatment resistant. The red vertical lines in each graph indicate the week that baloxavir-resistance first exceeds 20% of all new cases.

4.8 Pandemic scenarios

The results in this section correspond to pandemic scenarios in which the basic reproduction numbers (R_0) of the wild-type and resistant strains are set to 2.5 and 2.25, unless specified otherwise. The other model parameters are identical to the seasonal epidemic scenarios.

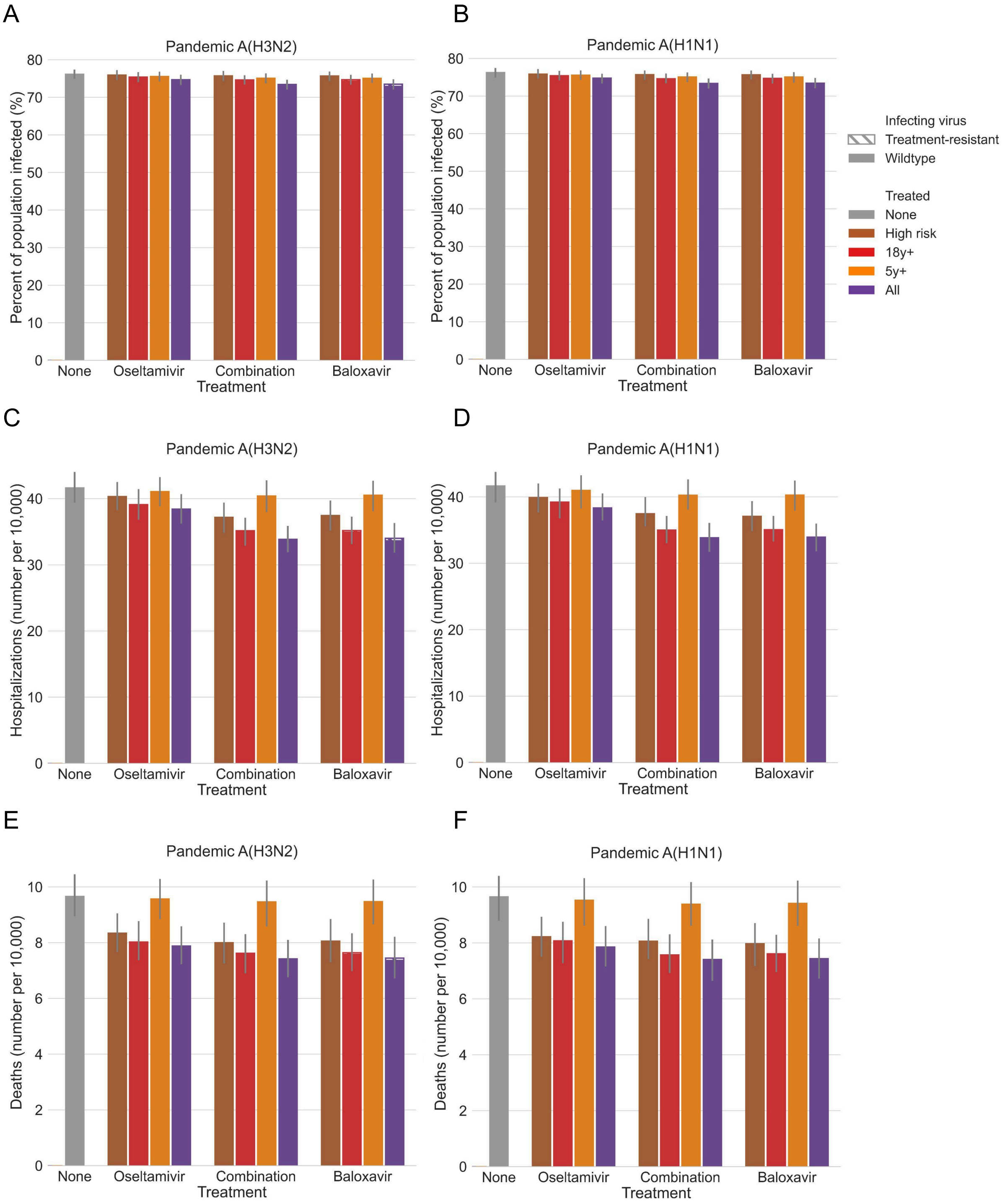

Figure S4.8. Impact of age-restricted treatment on the proportion of the population infected, hospitalized, or dying with wild-type influenza (solid) and treatment-resistant (hatched) influenza viruses. For (A,C,E) A(H3N2)-dominant pandemics or (B,D,F) A(H1N1)-dominant pandemics**.** Simulations assume reproduction numbers (R_0) of 2.5 for the wild-type virus and 2.25 for the baloxavir-resistant virus. Bar heights and gray lines indicate medians and 95% confidence intervals across 500 simulations.

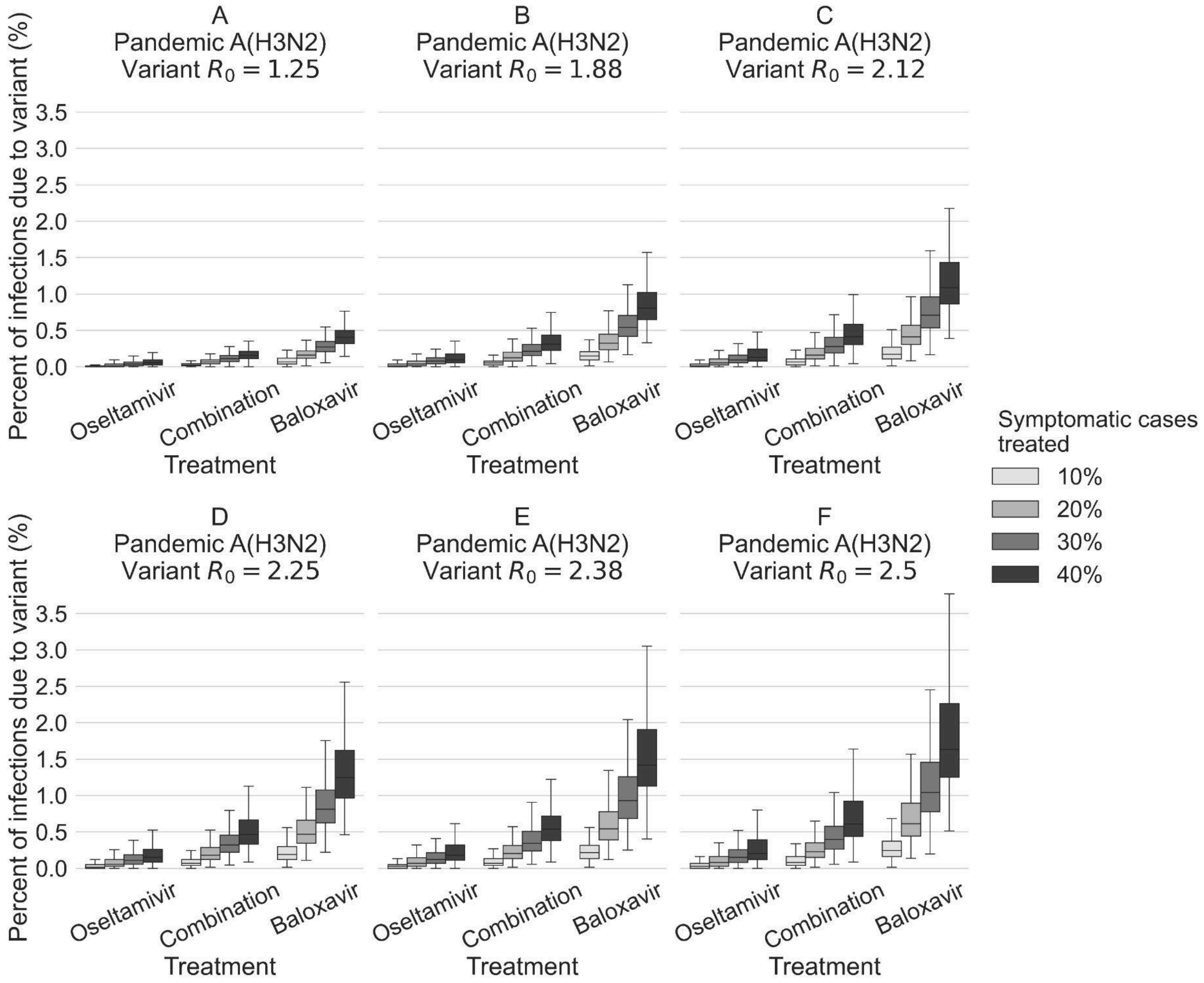

A
Pandemic A(H3N2)
Variant $R_0 = 1.25$
B
Pandemic A(H3N2)
Variant $R_0 = 1.88$
C
Pandemic A(H3N2)
Variant $R_0 = 2.12$
D
Pandemic A(H3N2)
Variant $R_0 = 2.25$
E
Pandemic A(H3N2)
Variant $R_0 = 2.38$
F
Pandemic A(H3N2)
Variant $R_0 = 2.5$
Percent of infections due to variant (%)
0.0
0.5
1.0
1.5
2.0
2.5
3.0
3.5
Oseltamivir
Combination
Baloxavir
Treatment
Symptomatic cases treated
10%
20%
30%
40%

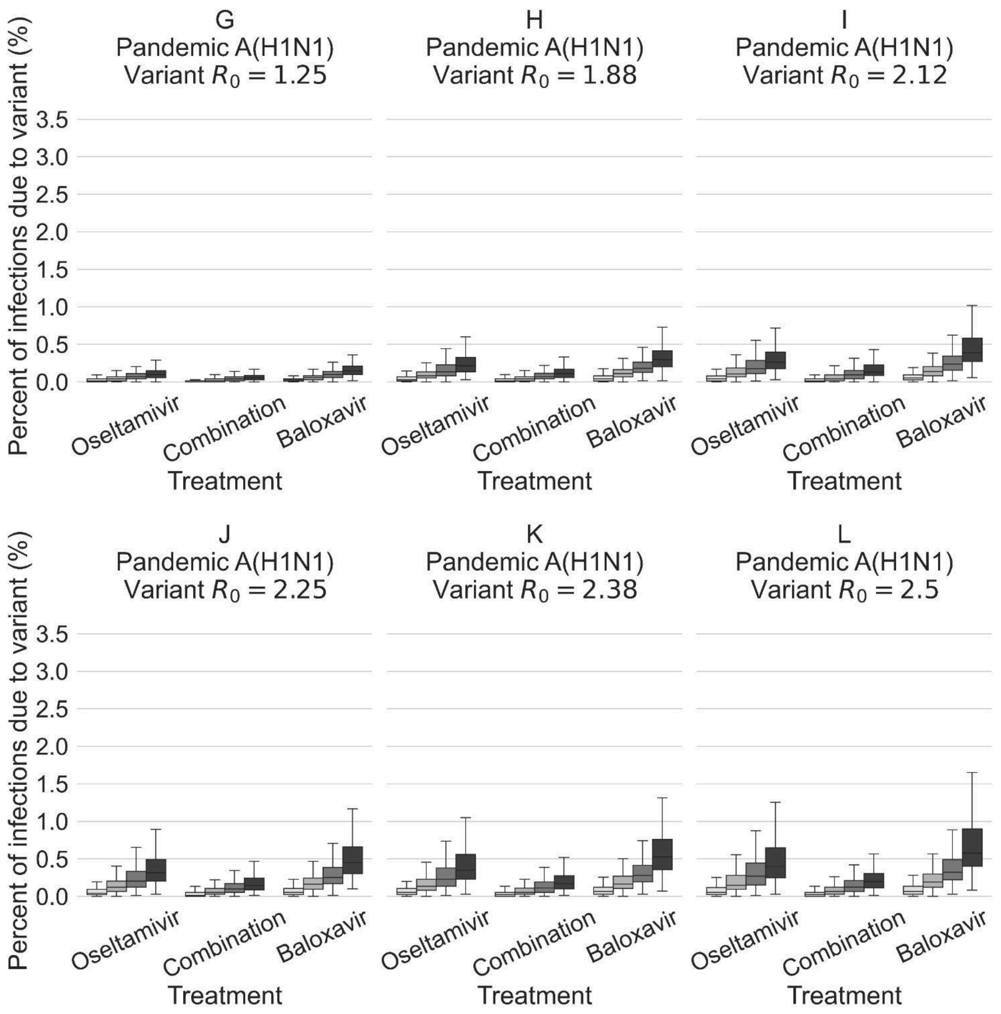

Figure S4.9. Impact of treatment on the proportion of all infections due to treatment-resistant strains. For (A, B, C, D, E, F) A(H3N2)-dominated pandemic events or (G, H, I, J, K, L) A(H1N1)-dominated pandemic events, depending on the fitness of the treatment-resistant strain. Combination therapy leads to baloxavir-resistant variant emergence only. The x-axes indicate the type of treatment administered; colors correspond to the proportion of symptomatic cases that receive treatment. All scenarios assume a wild-type R_0 of 2.5. The treatment-resistant R_0 differs as follows: (A, G) 50% of wild-type (1.25); (B, H) 75% of wild-type (1.88); (C, I) 85% of wild-type (2.12); (D, J) 90% of wild-type (2.25); (E, K) 95% of wild-type (2.38); (F, L) equal wild-type (2.5).

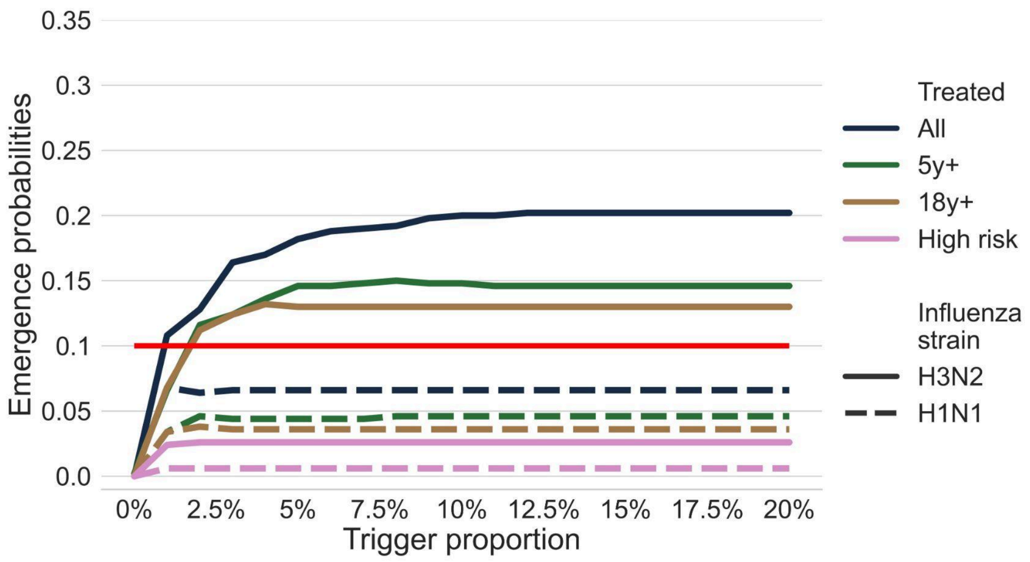

Figure S4.10. Probability of antiviral-resistant strains ever exceeding 20% of total weekly influenza incidence depending on the trigger for suspending treatment. This assumes that 20% of symptomatic cases in the specified group are treated with baloxavir in a pandemic scenario. The x-axis indicates the relative frequency of resistant infections at which treatment is suspended for the remainder of the season. The graph depicts results for both A(H3N2)-dominated (solid lines) and A(H1N1)-dominated (dashed lines) seasons, assuming a wild-type R_0=2.5 and treatment-resistant R_0=2.25. Probabilities are calculated as the proportion of simulations in which weekly incidence reaches 20% or more of total, across 500 simulations. The red solid line corresponds to a 10% chance that antiviral resistance will surpass 20% relative incidence sometime during the pandemic.

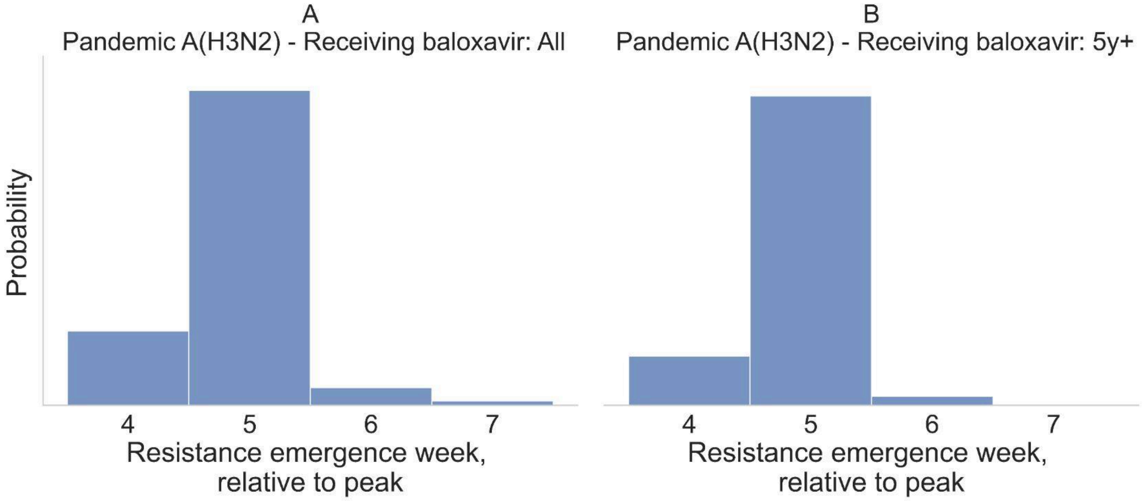

Figure S4.11. Timing of baloxavir-resistant emergence assuming that 20% of symptomatic cases are treated and that baloxavir treatment is suspended when resistance reaches 5% of relative incidence. All scenarios assume wild-type R_0=2.5 and treatment-resistant R_0=2.25, and an A(H3N2)-dominated pandemic. (A) Distribution of the first week where baloxavir-treatment resistant infections exceed 20% of incidence, assuming all treated patients receive baloxavir, and (B) only treated patients over age 5 receive baloxavir.

4.9 Sensitivity analysis: lower reproduction number

The results in this section correspond to epidemic scenarios where the basic reproduction number R_0 of the wild-type virus is lower than in our baseline scenario (1.12) and the R_0 of the antiviral-resistant viruses are 1.01, unless specified otherwise. The rest of the model parameters match the baseline scenarios. Because our initial results indicate that risk of baloxavir-resistance is significantly higher for A(H3N2) than A(H1N1), we focus our sensitivity analysis on an A(H3N2)-dominant season.

The qualitative results are similar to the baseline scenario. The probability that a treatment-resistant variant represents a 20% or more of weekly incidence remains similar (Figures S4.14 and S4.15), with a similar timing for emergence (Figure S4.16), and slightly less effective triggers (Figure S4.14). However, baloxavir treatment is expected to cause a larger proportional reduction in cases (compare Figure S4.12A to Figure S4.1A) and deaths (compare Figure S4.12B to Figure 2A)) as the effective reproduction number of the wild-type virus becomes closer to 1.0, thus reducing the final epidemic size. The risk of resistance emerging and representing a large fraction of total burden is similar (compare Figure S4.13B to Figure S4.4).

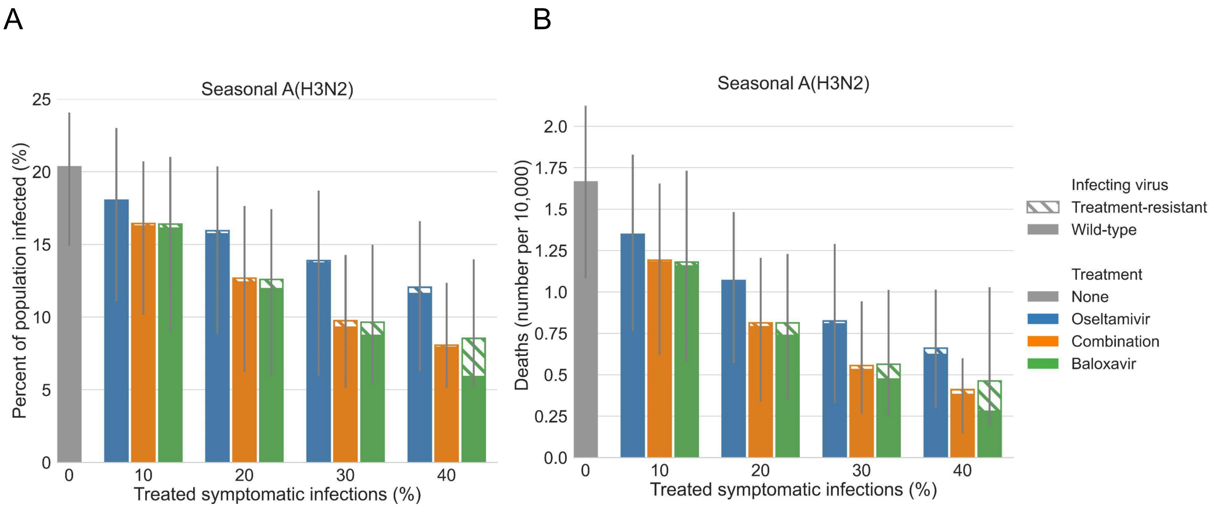

Figure S4.12. Projected impact of expanding treatment with oseltamivir, baloxavir or combination therapy for seasonal A(H3N2). This assumes a wild-type R_0=1.12 with treatment-resistant R_0=1.01. (A) Percent of the population infected with a wild-type (solid bar) or treatment-resistant (hatched bar) virus, based on 500 stochastic simulations. Bar heights indicate the median percent of the population infected, gray lines indicate 95% confidence intervals. The x-axes indicate the proportion of symptomatic cases that receive treatment; colors correspond to the type of treatment administered. (B) provides corresponding estimates for deaths per 10,000.

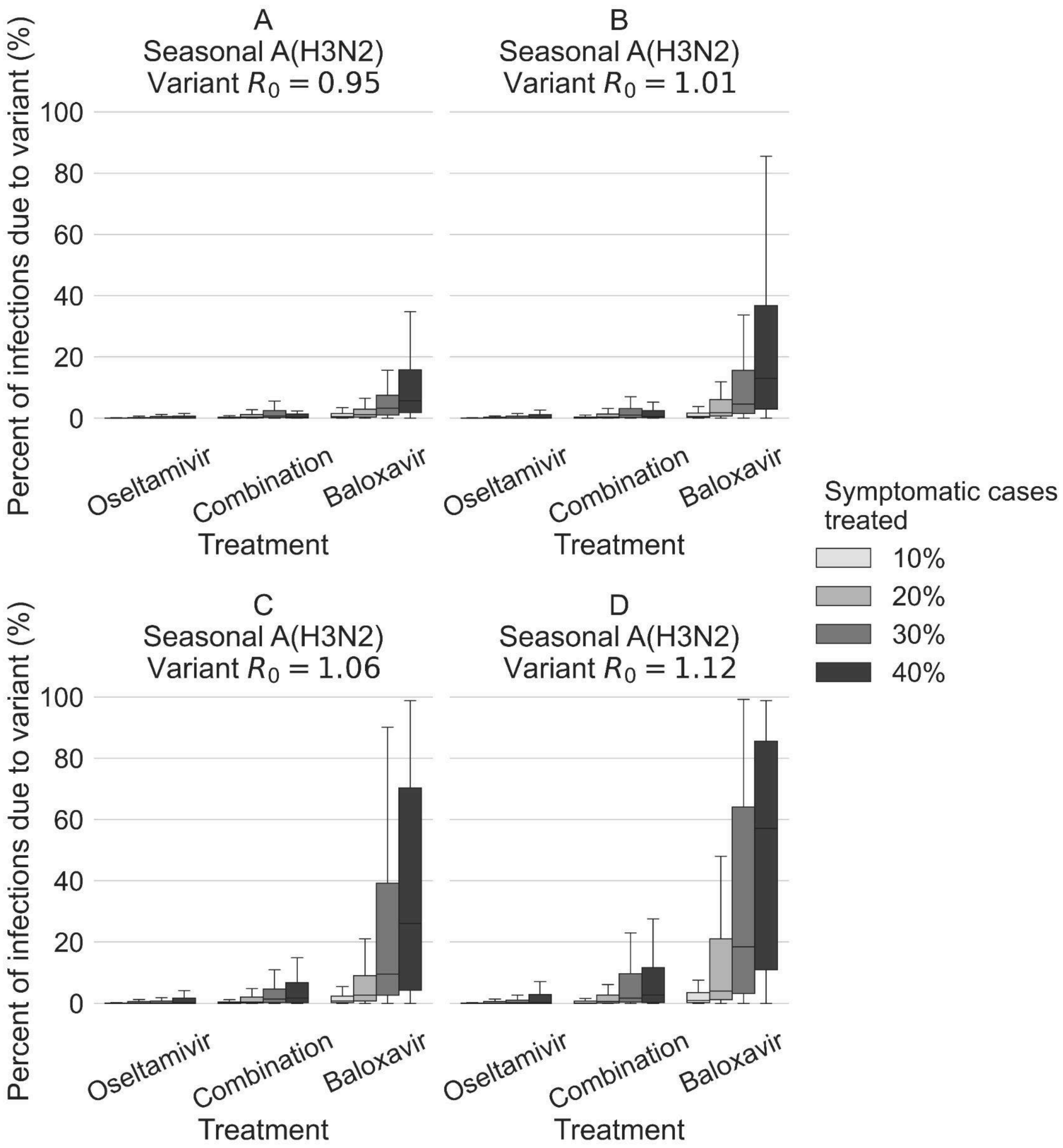

Figure S4.13. Impact of treatment on the proportion of all infections due to treatment-resistant variants during an A(H3N2)-dominated season, depending on the fitness of the treatment-resistant strain. Combination therapy leads to baloxavir-resistant variant emergence only. The x-axes indicate the type of treatment administered; colors correspond to the proportion of symptomatic cases that receive treatment. All scenarios assume a wild-type R_0 of 1.12. The treatment-resistant R_0 differs as follows: (A) 85% of wild-type (0.95); (B) 90% of wild-type (1.01); (C) 95% of wild-type (1.06); (D) equal to wild-type (1.12).

A

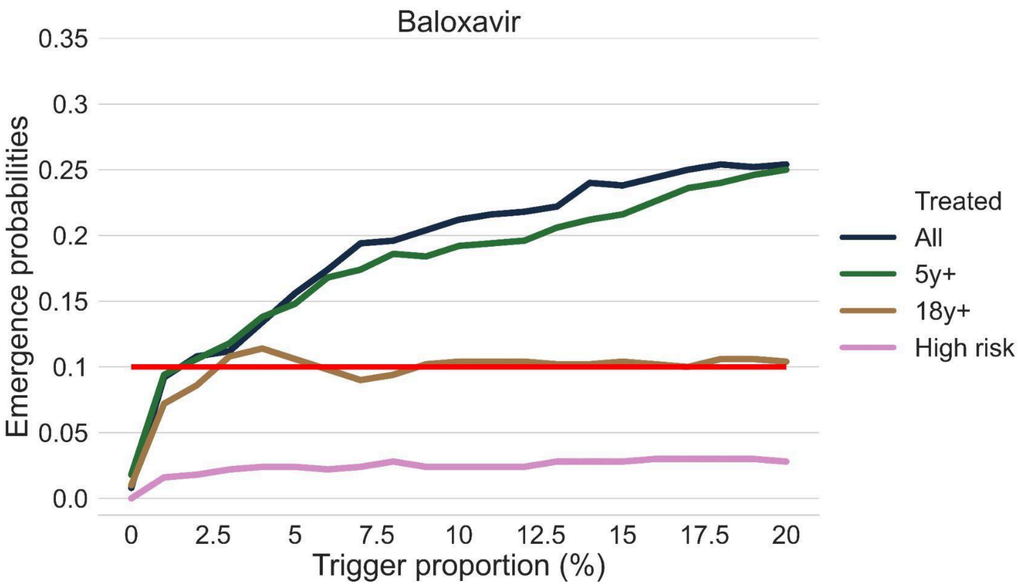

B

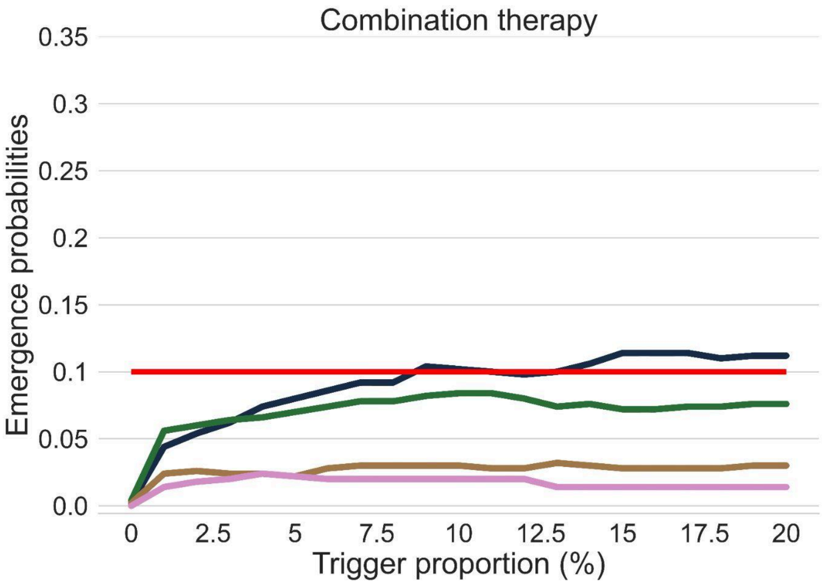

Figure S4.14. Probability of antiviral-resistant variants ever representing over 20% of total weekly incidence as a function of triggers on the share of weekly incidence to stop antiviral allocation. Probability of antiviral-resistant strains ever exceeding 20% of total weekly influenza incidence depending on the trigger for suspending treatment, assuming that 20% of symptomatic cases in the specified group are treated with either (A) baloxavir, or (B) combination therapy. The x-axis indicates the relative frequency of resistant infections at which treatment is suspended for the remainder of the season. This assumes a wild-type R_0=1.12 with treatment-resistant R_0=1.01 and an A(H3N2)-dominated season. Probabilities are calculated as the proportion of simulations in which weekly incidence reaches 20% or more of total, across 500 simulations. The red solid line corresponds to a 10% chance that antiviral resistance will surpass 20% relative incidence sometime during the influenza season.

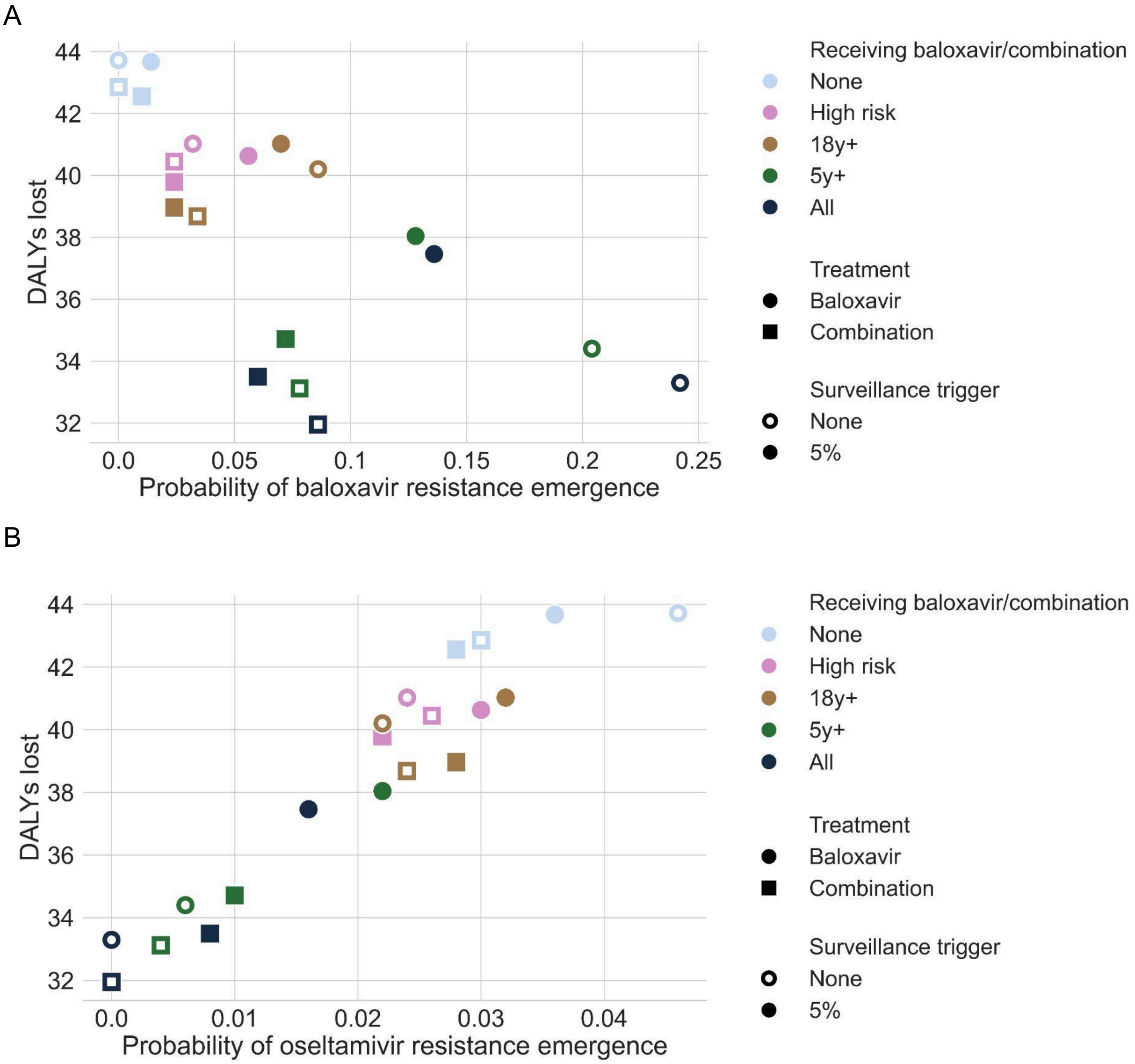

Figure S4.15. Trade-off between public health benefits of antiviral treatment (DALYs lost per 10,000 individuals) and the risk that (A) baloxavir-resistant viruses and (B) oseltamivir-resistant viruses will emerge and spread. Emergence is defined as the probability of exceeding 20% of incident infections anytime during the season**.** This assumes a wild-type R_0=1.12 with treatment-resistant R_0=1.01 and an A(H3N2)-dominated season. Each of the 20 antiviral treatment strategies assumes that 20% of symptomatic cases receive treatment and specifies the following treated subpopulations to receive either baloxavir (circles) or combination therapy (squares) rather than oseltamivir: none (light blue), high risk individuals only (pink), adults over 18y (brown), children and adults over 5y (green), or all (black). Each strategy also either does (filled symbols) or does not (open symbols) include a surveillance-based trigger for suspending baloxavir and combination therapy when baloxavir-resistant viruses surpass 5% relative incidence, and for suspending oseltamivir when oseltamivir-resistant viruses surpass 5% relative incidence. The estimates are medians across 500 stochastic simulations.

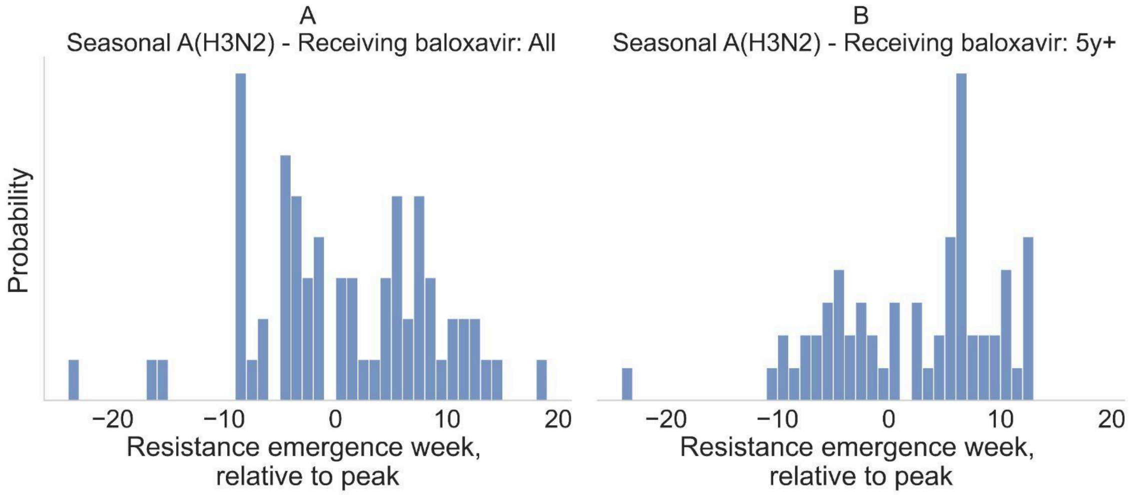

Figure S4.16. Timing of baloxavir-resistant emergence assuming that 20% of symptomatic cases are treated and that baloxavir treatment is suspended when resistance reaches 5% of relative incidence. All scenarios assume wild-type R_0=1.12 and treatment-resistant R_0=1.01, and an A(H3N2)-dominated epidemic. (A) Distribution of the first week where baloxavir-treatment resistant infections exceed 20% of incidence, assuming all treated patients receive baloxavir, and (B) only treated patients over age 5 receive baloxavir (younger patients receive oseltamivir).

4.10 Sensitivity analysis: Impact of expanded antiviral treatment on disease burden when antiviral-resistant variant has same transmission as wild-type

The figures below show results to those shown in Figures 2, S4.1, and S4.2 when we assume no loss of relative fitness for the antiviral-resistant variant. In this case we see that using combination therapy reduces total disease burden to a greater extent than baloxavir, with the comparative benefits becoming greater as the proportion of population treated increases.

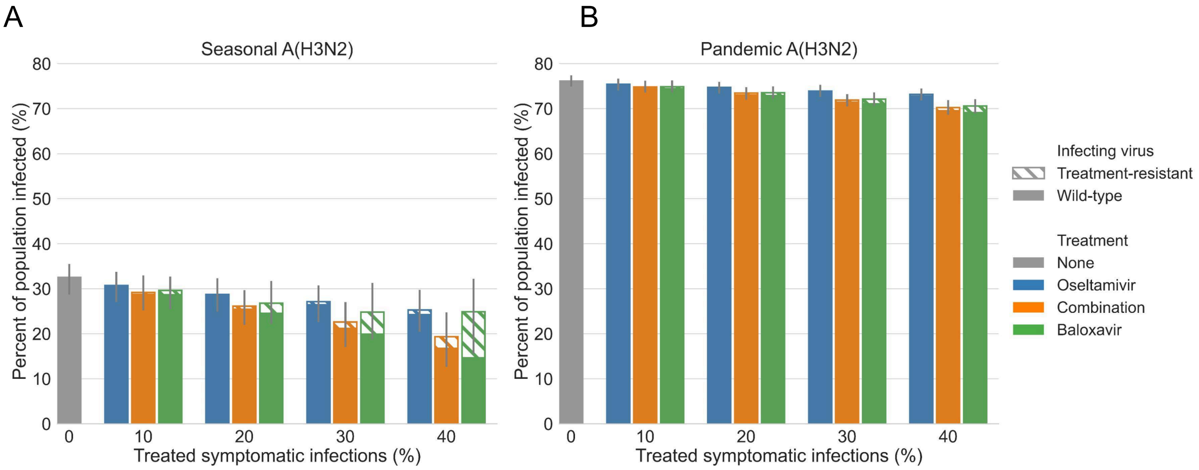

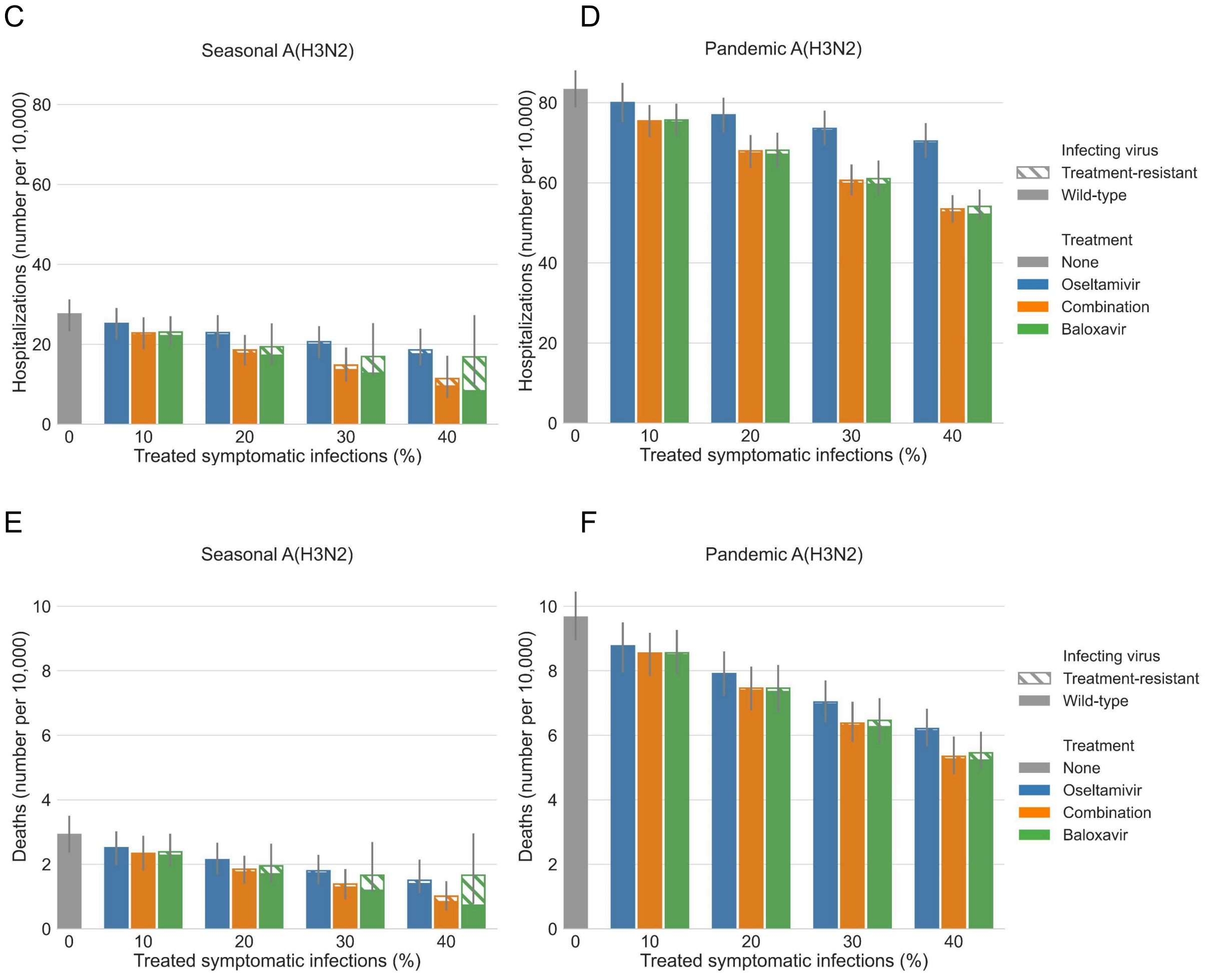

Figure S4.17. Projected impact of expanding treatment with oseltamivir, baloxavir or combination therapy for seasonal and pandemic influenza A(H3N2) when wild-type and treatment-resistant variant viruses have the same transmission fitness. For (A) a seasonal epidemic where wild-type and treatment-resistant viruses with R_0=1.28 and (B) a pandemic virus with R_0=2.5, bar heights indicate the median percent of the population infected with a wild-type (solid bar) or treatment-resistant (hatched bar) virus, based on 500 stochastic simulations. Gray lines indicate 95% confidence intervals. The x-axes indicate the proportion of symptomatic cases that receive treatment; colors correspond to the type of treatment administered. (C) and (D) provide corresponding estimates for hospitalizations per 10,000; (E) and (F) provide corresponding estimates for deaths per 10,000.

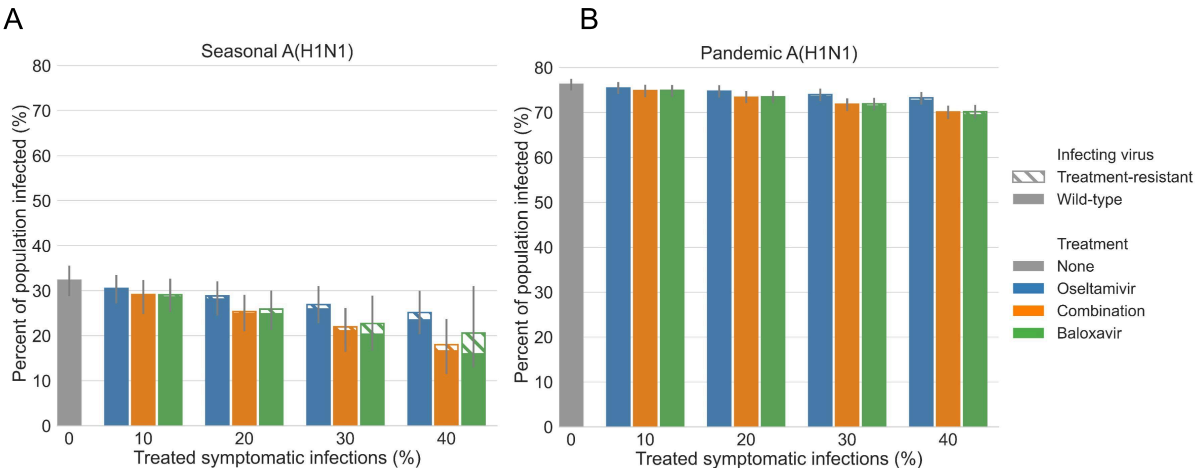
A
Seasonal A(H1N1)
B
Pandemic A(H1N1)
Percent of population infected (%)
Treated symptomatic infections (%)
0
10
20
30
40
50
60
70
80
Infecting virus
Treatment-resistant
Wild-type
Treatment
None
Oseltamivir
Combination
Baloxavir

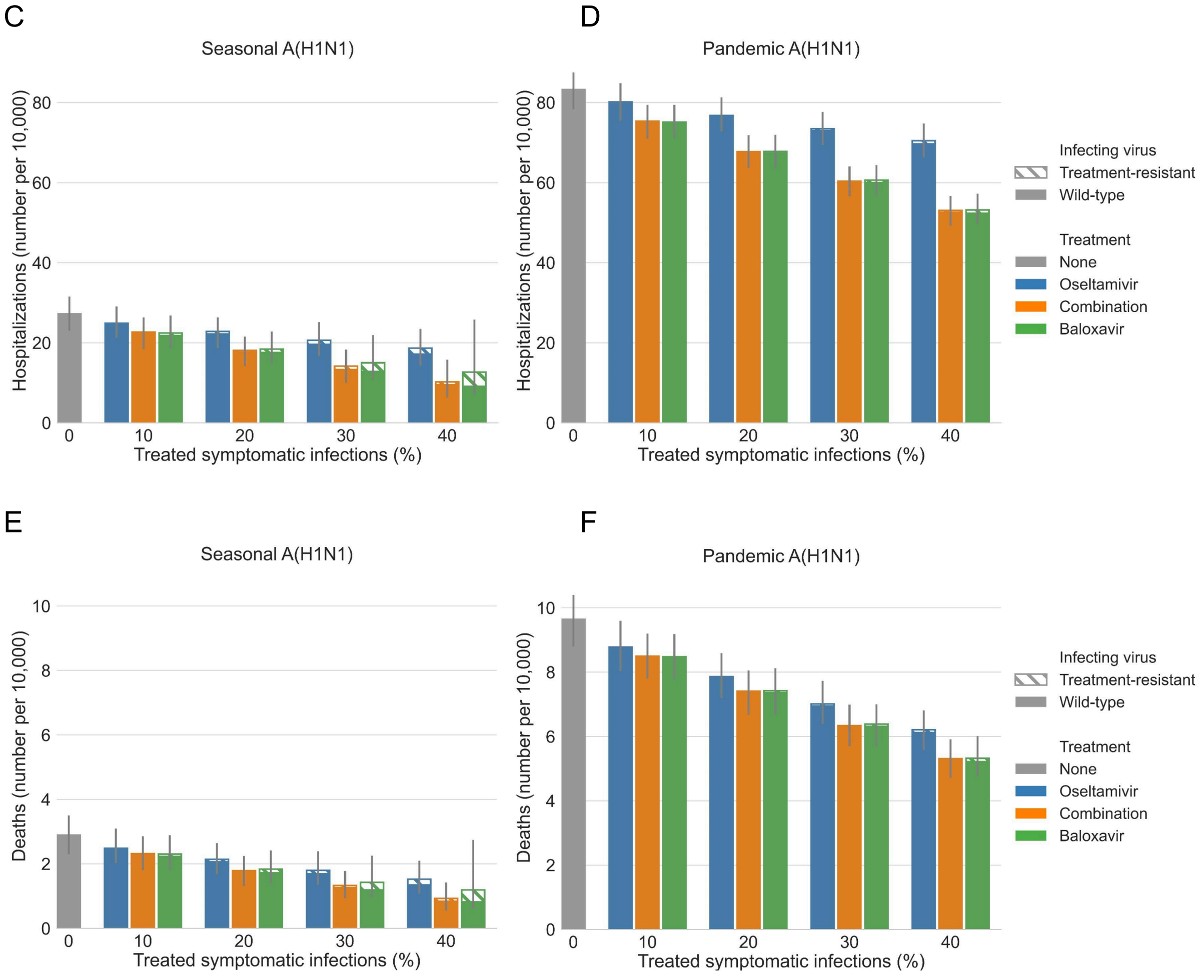

Figure S4.18. Projected impact of expanding treatment with oseltamivir, baloxavir or combination therapy for seasonal and pandemic influenza A(H1N1) when wild-type and treatment-resistant variant viruses have the same transmission fitness. For (A) a seasonal epidemic where wild-type and treatment-resistant viruses with R_0=1.28 and (B) a pandemic virus with R_0=2.5, bar heights indicate the median percent of the population infected with a wild-type (solid bar) or treatment-resistant (hatched bar) virus, based on 500 stochastic simulations. Gray lines indicate 95% confidence intervals. The x-axes indicate the proportion of symptomatic cases that receive treatment; colors correspond to the type of treatment administered. (C) and (D) provide corresponding estimates for hospitalizations per 10,000; (E) and (F) provide corresponding estimates for deaths per 10,000.

4.11 Sample epidemiological curves and corresponding resistance emergence probability under various treatment strategies

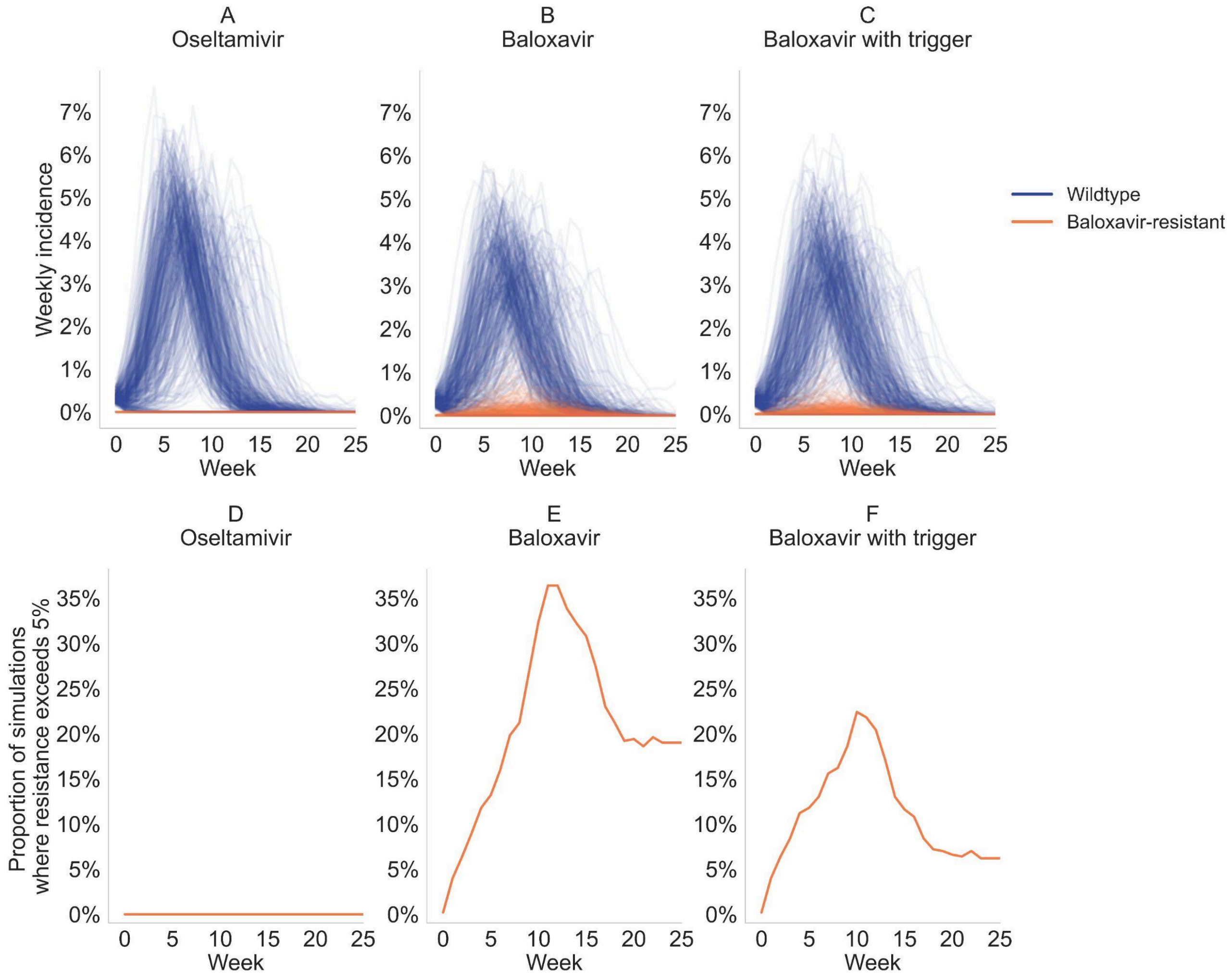

Figure S4.19. Projected influenza-related incidence and baloxavir resistance probability during a typical influenza A(H3N2)-dominated season. This assumes 20% of symptomatic cases receive treatment. Treated individuals receive (A) oseltamivir throughout the season, (B) baloxavir throughout the season, or (C) baloxavir until baloxavir-resistant viruses surpass 5% relative incidence and oseltamivir thereafter. Lines correspond to single simulations in which the cumulative infections fell within the IQR; blue and orange indicating infections attributable to wild-type or resistant viruses, respectively. (D, E, F) Proportion of simulations for which baloxavir-resistant viruses surpass 5% relative incidence each week for the treatment strategy displayed in the plot just above. Simulations assume reproduction numbers (R_0) of 1.28 for the wild-type virus and 1.15 for the baloxavir-resistant virus.

References

1. Lampejo T. Influenza and antiviral resistance: an overview. Eur J Clin Microbiol Infect Dis. 2020;39: 1201–1208. doi:10.1007/s10096-020-03840-9

2. Gubareva LV, Fry AM. Baloxavir and Treatment-Emergent Resistance: Public Health Insights and Next Steps. J Infect Dis. 2020;221: 337–339. doi:10.1093/infdis/jiz245

3. Du Z, Nugent C, Galvani AP, Krug RM, Meyers LA. Modeling mitigation of influenza epidemics by baloxavir. Nat Commun. 2020;11: 2750. doi:10.1038/s41467-020-16585-y

4. Baccam P, Beauchemin C, Macken CA, Hayden FG, Perelson AS. Kinetics of influenza A virus infection in humans. J Virol. 2006;80: 7590–7599. doi:10.1128/JVI.01623-05

5. A Study of S-033188 (Baloxavir Marboxil) Compared With Placebo or Oseltamivir in Otherwise Healthy Patients With Influenza (CAPSTONE 1). [cited 5 Oct 2022]. Available: https://beta.clinicaltrials.gov/study/NCT02954354

6. Hayden FG, Sugaya N, Hirotsu N, Lee N, de Jong MD, Hurt AC, et al. Baloxavir Marboxil for Uncomplicated Influenza in Adults and Adolescents. N Engl J Med. 2018;379: 913–923. doi:10.1056/NEJMoa1716197

7. Study to Assess the Safety, Pharmacokinetics, and Efficacy of Baloxavir Marboxil in Healthy Pediatric Participants With Influenza-Like Symptoms. [cited 5 Oct 2022]. Available: https://beta.clinicaltrials.gov/study/NCT03629184

8. Baker J, Block SL, Matharu B, Burleigh Macutkiewicz L, Wildum S, Dimonaco S, et al. Baloxavir Marboxil Single-dose Treatment in Influenza-infected Children: A Randomized, Double-blind, Active Controlled Phase 3 Safety and Efficacy Trial (miniSTONE-2). Pediatr Infect Dis J. 2020;39: 700–705. doi:10.1097/INF.0000000000002747

9. Whitley RJ, Hayden FG, Reisinger KS, Young N, Dutkowski R, Ipe D, et al. Oral oseltamivir treatment of influenza in children. Pediatr Infect Dis J. 2001;20: 127–133. doi:10.1097/00006454-200102000-00002

10. Hirotsu N, Sakaguchi H, Sato C, Ishibashi T, Baba K, Omoto S, et al. Baloxavir Marboxil in Japanese Pediatric Patients With Influenza: Safety and Clinical and Virologic Outcomes. Clin Infect Dis. 2020;71: 971–981. doi:10.1093/cid/ciz908

11. Lessler J, Reich NG, Brookmeyer R, Perl TM, Nelson KE, Cummings DAT. Incubation periods of acute respiratory viral infections: a systematic review. Lancet Infect Dis. 2009;9: 291–300. doi:10.1016/S1473-3099(09)70069-6

12. Handel A, Longini IM Jr, Antia R. Neuraminidase inhibitor resistance in influenza: assessing the danger of its generation and spread. PLoS Comput Biol. 2007;3: e240. doi:10.1371/journal.pcbi.0030240

13. van Laarhoven PJM, Aarts EHL. Simulated annealing. In: van Laarhoven PJM, Aarts EHL, editors. Simulated Annealing: Theory and Applications. Dordrecht: Springer Netherlands; 1987. pp. 7–15. doi:10.1007/978-94-015-7744-1_2

14. scipy.optimize.dual_annealing — SciPy v1.9.1 Manual. [cited 5 Oct 2022]. Available:

https://docs.scipy.org/doc/scipy/reference/generated/scipy.optimize.dual_annealing.html

15. Meyers LA, Pourbohloul B, Newman MEJ, Skowronski DM, Brunham RC. Network theory and SARS: predicting outbreak diversity. J Theor Biol. 2005;232: 71–81. doi:10.1016/j.jtbi.2004.07.026

16. Tsang TK, Cowling BJ, Fang VJ, Chan K-H, Ip DKM, Leung GM, et al. Influenza A Virus Shedding and Infectivity in Households. J Infect Dis. 2015;212: 1420–1428. doi:10.1093/infdis/jiv225

17. Suess T, Remschmidt C, Schink SB, Schweiger B, Heider A, Milde J, et al. Comparison of shedding characteristics of seasonal influenza virus (sub)types and influenza A(H1N1)pdm09; Germany, 2007-2011. PLoS One. 2012;7: e51653. doi:10.1371/journal.pone.0051653

18. Redlberger-Fritz M, Hirk S, Buchinger D, Haberl R, Hell M, Perkmann-Nagele N, et al. Distinct differences in clinical manifestation and viral laboratory parameters between children and adults with influenza A(H1N1)pdm09 infection--a retrospective comparative analysis. J Med Virol. 2014;86: 1048–1055. doi:10.1002/jmv.23912

19. Uehara T, Hayden FG, Kawaguchi K, Omoto S, Hurt AC, De Jong MD, et al. Treatment-Emergent Influenza Variant Viruses With Reduced Baloxavir Susceptibility: Impact on Clinical and Virologic Outcomes in Uncomplicated Influenza. J Infect Dis. 2020;221: 346–355. doi:10.1093/infdis/jiz244

20. Kumar D, Ison MG, Mira J-P, Welte T, Hwan Ha J, Hui DS, et al. Combining baloxavir marboxil with standard-of-care neuraminidase inhibitor in patients hospitalised with severe influenza (FLAGSTONE): a randomised, parallel-group, double-blind, placebo-controlled, superiority trial. Lancet Infect Dis. 2022;22: 718–730. doi:10.1016/S1473-3099(21)00469-2

21. Lina B, Boucher C, Osterhaus A, Monto AS, Schutten M, Whitley RJ, et al. Five years of monitoring for the emergence of oseltamivir resistance in patients with influenza A infections in the Influenza Resistance Information Study. Influenza Other Respi Viruses. 2018;12: 267–278. doi:10.1111/irv.12534

22. Yokoyama T, Sakaguchi H, Ishibashi T, Shishido T, Piedra PA, Sato C, et al. Baloxavir Marboxil 2% Granules in Japanese Children With Influenza: An Open-label Phase 3 Study. Pediatr Infect Dis J. 2020;39: 706–712. doi:10.1097/INF.0000000000002748

23. Sonoyama T, Sakaguchi H, Koshimichi H, Noshi T, Tsuchiya K, Uehara T. Open-label study of the safety, pharmacokinetics, and effectiveness of a 2 mg/kg dose of baloxavir marboxil 2% granules in children <20 kg with influenza. J Infect Chemother. 2021;27: 1223–1229. doi:10.1016/j.jiac.2021.05.009

24. US Census Bureau. Public Use Microdata Sample (PUMS). [cited 28 Sep 2022]. Available: https://www.census.gov/programs-surveys/acs/microdata.html

25. Number and percentage distribution of public elementary and secondary schools and enrollment, by level, type, and enrollment size of school: 2015-16, 2016-17, and 2017-18. [cited 5 Oct 2022]. Available: https://nces.ed.gov/programs/digest/d19/tables/dt19_216.40.asp?current=yes

26. Enrollment in public elementary and secondary schools, by level and grade: Selected years, fall 1980 through fall 2030. [cited 5 Oct 2022]. Available: https://nces.ed.gov/programs/digest/d21/tables/dt21_203.10.asp?current=yes

27. Private elementary and secondary school enrollment and private enrollment as a percentage of total enrollment in public and private schools, by region and grade level. [cited 5 Oct 2022]. Available: https://nces.ed.gov/programs/digest/d21/tables/dt21_205.10.asp?current=yes

28. Staff employed in public elementary and secondary school systems, by type of assignment: Selected years, 1949-50 through fall 2019. [cited 5 Oct 2022]. Available: https://nces.ed.gov/programs/digest/d21/tables/dt21_213.10.asp?current=yes

29. Civilian labor force participation rate by age, sex, race, and ethnicity. 8 Sep 2022 [cited 5 Oct 2022]. Available: https://www.bls.gov/emp/tables/civilian-labor-force-participation-rate.htm

30. E-16. Unemployment rates by age, sex, race, and Hispanic or Latino ethnicity. 8 Jul 2022 [cited 5 Oct 2022]. Available: https://www.bls.gov/web/empsit/cpsee_e16.htm

31. US Business Firmographics – Company Size. In: NAICS Association [Internet]. [cited 5 Oct 2022]. Available: https://www.naics.com/business-lists/counts-by-company-size/

32. Mistry D, Litvinova M, Pastore Y Piontti A, Chinazzi M, Fumanelli L, Gomes MFC, et al. Inferring high-resolution human mixing patterns for disease modeling. Nat Commun. 2021;12: 323. doi:10.1038/s41467-020-20544-y

33. Pasco RF, Fox SJ, Johnston SC, Pignone M, Meyers LA. Estimated Association of Construction Work With Risks of COVID-19 Infection and Hospitalization in Texas. JAMA Netw Open. 2020;3: e2026373. doi:10.1001/jamanetworkopen.2020.26373

34. Ip DKM, Lau LLH, Leung NHL, Fang VJ, Chan K-H, Chu DKW, et al. Viral Shedding and Transmission Potential of Asymptomatic and Paucisymptomatic Influenza Virus Infections in the Community. Clin Infect Dis. 2017;64: 736–742. doi:10.1093/cid/ciw841

35. Past Seasons Estimated Influenza Disease Burden. 1 Oct 2020 [cited 30 Sep 2022]. Available: https://www.cdc.gov/flu/about/burden/past-seasons.html

36. Biggerstaff M, Cauchemez S, Reed C, Gambhir M, Finelli L. Estimates of the reproduction number for seasonal, pandemic, and zoonotic influenza: a systematic review of the literature. BMC Infect Dis. 2014;14: 480. doi:10.1186/1471-2334-14-480

37. Loeb M, Singh PK, Fox J, Russell ML, Pabbaraju K, Zarra D, et al. Longitudinal study of influenza molecular viral shedding in Hutterite communities. J Infect Dis. 2012;206: 1078–1084. doi:10.1093/infdis/jis450

38. Hanslik T, Boelle P-Y, Flahault A. Preliminary estimation of risk factors for admission to intensive care units and for death in patients infected with A(H1N1)2009 influenza virus, France, 2009-2010. PLoS Curr. 2010;2: RRN1150. doi:10.1371/currents.rrn1150

39. Van Kerkhove MD, Vandemaele KAH, Shinde V, Jaramillo-Gutierrez G, Koukounari A, Donnelly CA, et al. Risk factors for severe outcomes following 2009 influenza A (H1N1)

infection: a global pooled analysis. PLoS Med. 2011;8: e1001053. doi:10.1371/journal.pmed.1001053

40. Hsu J, Santesso N, Mustafa R, Brozek J, Chen YL, Hopkins JP, et al. Antivirals for treatment of influenza: a systematic review and meta-analysis of observational studies. Ann Intern Med. 2012;156: 512–524. doi:10.7326/0003-4819-156-7-201204030-00411

41. Neuberger E, Wallick C, Chawla D, Castro R de C. Baloxavir vs oseltamivir: reduced utilization and costs in influenza. Am J Manag Care. 2022;28: e88–e95. doi:10.37765/ajmc.2022.88786

42. Disability-adjusted life years (DALYs). [cited 5 Oct 2022]. Available: https://www.who.int/data/gho/indicator-metadata-registry/imr-details/158

43. Sah P, Medlock J, Fitzpatrick MC, Singer BH, Galvani AP. Optimizing the impact of low-efficacy influenza vaccines. Proc Natl Acad Sci U S A. 2018;115: 5151–5156. doi:10.1073/pnas.1802479115

44. Arias E, Xu J. United States Life Tables, 2019. Natl Vital Stat Rep. 2022;70: 1–59. doi:10.15620/cdc:113096

45. Cowling B. Phase III CENTERSTONE study of single-dose baloxavir marboxil▼ for the reduction of transmission of influenza in households - Options-XII-2024-presentation-monto-phase-III-CENTERSTONE-study-of-single-dose.pdf. Roche; 30 Sep 2024 [cited 23 Dec 2024]. Available: https://medically.roche.com/global/en/infectious-disease/options-xii-2024/medical-material/Options-XII-2024-presentation-monto-phase-III-CENTERSTONE-study-of-single-dose-pdf.html
